# Contribution to the knowledge of the clown beetle fauna of Lebanon, with a key to all species (Coleoptera, Histeridae)

**DOI:** 10.3897/zookeys.960.50186

**Published:** 2020-08-17

**Authors:** Salman Shayya, Tomáš Lackner

**Affiliations:** 1 Faculty of Health Sciences, American University of Science and Technology, Beirut, Lebanon American University of Science and Technology Beirut Lebanon; 2 Bavarian State Collection of Zoology, Münchhausenstraße 21, 81247 Munich, Germany Bavarian State Collection of Zoology Munich Germany

**Keywords:** Coleoptera, faunistics, Lebanon, Histeridae, Histerinae, Saprininae, Tribalinae

## Abstract

The occurrence of histerids in Lebanon has received little specific attention. Hence, an aim to enrich the knowledge of this coleopteran family through a survey across different Lebanese regions in this work. Seventeen species belonging to the genera *Atholus* Thomson, 1859, *Hemisaprinus* Kryzhanovskij, 1976, *Hister* Linnaeus, 1758, *Hypocacculus* Bickhardt, 1914, *Margarinotus* Marseul, 1853, *Saprinus* Erichson, 1834, *Tribalus* Erichson, 1834, and *Xenonychus* Wollaston, 1864 were recorded. Specimens were sampled mainly with pitfall traps baited with ephemeral materials like pig dung, decayed fish, and pig carcasses. Several species were collected by sifting soil detritus, sand cascading, and other specialized techniques. Six newly recorded species for the Lebanese fauna are the necrophilous *Hister sepulchralis* Erichson, 1834, *Hemisaprinus subvirescens* (Ménétriés, 1832), Saprinus (Saprinus) externus (Fischer von Waldheim, 1823), Saprinus (Saprinus) figuratus Marseul, 1855, and Saprinus (Saprinus) niger (Motschulsky, 1849) all associated with rotting fish and dung, and the psammophilous *Xenonychus tridens* (Jacquelin du Val, 1853). With the exception of *Hister sepulchralis*, all these taxa belong to the Saprininae subfamily. A most likely undescribed species of Tribalus (Tribalus) (Tribalinae) has also been collected in detritus at wet places near rivers in Lebanon. Because of the complexity of the genus *Tribalus*, with possible numerous new species present in the circum-Mediterranean area, the Lebanese species is not described herein, pending a revision of the genus. This study advocates further research aimed at improving taxonomic and ecological knowledge of this coleopteran family in Lebanon. The number of Histeridae species currently known from Lebanon stands at 41; a key to all species including images is included.

## Introduction

The interesting biodiversity of Lebanon is due to its complex topography and altitudinal diversity and its location at the eastern rim of the Mediterranean Sea (Khater and El-Hajj 2012). According to Khater and El-Hajj (2012), there is a need to complete the assessment of biodiversity in various biological sections, especially invertebrates, which includes in its vast majority the insects. The latter occupy most ecological niches and are considered very important to the dynamics of natural ecosystems’ structure and function (Fagundes et al. 2011; Cajaiba et al. 2017). Preserving their abundance and diversity should constitute a prime conservation priority. Coleoptera is the largest hexapodan order with ca. 400,000 described species worldwide, which comprise 40% of all described insect species (Fagundes et al. 2011; Gimmel and Ferro 2018). Carrion, animal droppings, dead wood, bird nests, mushrooms, and ant and termite nests are examples of such microhabitats (Bajerlein 2009; Barbosa and Vasconcelos 2018). The physicochemical conditions of these microhabitats are fleeting and rapidly changing, but they are rich in organic matter and colonized by insects of various guilds: coprophagous or necrophagous species, parasitoids and predators (Bajerlein et al. 2011). Among the beetle fauna associated with cadavers and animal droppings, predation is the main force structuring communities (Goff 2009; Bajerlein et al. 2011). Many Histeridae specialize on these microhabitats and are considered predictable components of carrion and dung communities (Bajerlein et al. 2011). In addition, histerids can be found under bark, in sand, in the galleries of wood-boring insects, and as important predators in stored products (Polat and Yıldırım 2017).

Histeridae may fulfil important practical roles (Kovarik and Caterino 2016). Many Histeridae adults and larvae are necrophilous, feeding mainly on dipteran larvae present on carrion or dung (Caneparo et al. 2017; Szelecz et al. 2018). Some are general predators of mites, insects and insect larvae, while other species are specific feeders on larvae of a single insect species (Szelecz et al. 2018). During carrion decomposition, histerids arrive in large numbers when fly larvae are abundant, i.e., during the active decay and early advanced decay stages (Caneparo et al. 2017; Shayya et al. 2018; Szelecz et al. 2018). Since the predatory taxa are abundant at different stages of decomposition, the knowledge of both their occurrence and the rates of decomposition aids in estimating the minimum postmortem interval PMI_min_ on the basis of entomofaunal succession (Caneparo et al. 2017). The latter method could be the only accessible tool for estimating the PMI_min_ when the PMI is longer than several months or even years (Amendt et al. 2011). Other predatory species of Histeridae have been introduced to augment control of dung-breeding flies (Davis 1994).

Histeridae (clown beetles) contain 4260 species and 400 genera, grouped in nine subfamilies (Mazur 2011; Caterino and Tishechkin 2014; Lackner 2015; Zhou et al. 2020). Our current paper treats only six subfamilies: Abraeinae Macleay, 1819; Dendrophilinae Reitter, 1909; Histerinae Gyllenhal, 1808; Onthophilinae Macleay, 1819; Saprininae Blanchard, 1845 and Tribalinae Bickhardt, 1914. Saprininae and Histerinae contain most forensically-relevant taxa. Saprininae are a moderately large subfamily distributed worldwide (73 genera and subgenera, > 620 species (Mazur 2011; Lackner and Tarasov 2019). In the past ten years, this subfamily has been intensively studied with respect to their phylogeny (Lackner 2014a; Lackner and Tarasov 2019), zoogeographical distribution, taxonomy, morphology, and biology (e.g., Shayya et al. 2018; Lackner et al. 2019). Unfortunately, the rest of the subfamilies present in Lebanon have yet to witness attention from a phylogenetic standpoint. In the Palaearctic Region, 357 species of the Saprininae, 220 of Histerinae, 115 of Dendrophilinae, 101 of Abraeinae, 31 of Onthophilinae, and 31 species of Tribalinae, respectively, have been reported hitherto (Lackner et al. 2015).

Regarding the Saprininae, 23 species are known so far from Lebanon; three belonging to *Chalcionellus* Reichardt, 1932, one to *Gnathoncus* Jacquelin du Val, 1857 one to *Xenonychus* Wollaston, 1864, two to *Hypocacculus* Bickhardt, 1914, one to *Hypocaccus* C.G. Thomson, 1867 and 15 species belonging to *Saprinus* Erichson, 1834 (Lackner et al. 2015; Shayya et al. 2018; present study). Thirteen species of *Saprinus* have been collected on decomposing carrion from Lebanon; eight of them, as well as Hypocacculus (Hypocacculus) metallescens (Erichson, 1834) have only recently been recorded for the fauna of the country (Shayya et al. 2018).

Within the Histerinae, eleven species are currently known from Lebanon; one belonging to *Atholus* Thomson, 1859, one to *Eudiplister* Reitter, 1909, two to *Hister* Linnaeus, 1758, three to *Margarinotus* Marseul, 1853 (all Histerini), one species belonging to otherwise oriental genus *Notodoma* Lacordaire, 1854 (Exosternini), two species of *Platyliste*r Lewis, 1892, and one species of *Platysoma* Leach, 1817 (Platysomatini) (Lackner et al. 2015; Shayya et al. 2018). *Atholus duodecimstriatus duodecimstriatus* (Schrank, 1781), Margarinotus (Ptomister) brunneus (Fabricius, 1775), and Margarinotus (Grammostethus) ruficornis (Grimm, 1852) were recently newly recorded for the Lebanese fauna (Shayya et al. 2018). Within Dendrophilinae, two species of the genus *Abraeomorphus* Reitter, 1886, are known from Lebanon, while from Abraeinae, only *Stenopleurum rothi* (Rosenhauer, 1856) is recorded from the country. Two species of *Onthophilus* Leach, 1817 are known from Lebanon within Onthophilinae.

No representative of the subfamily Tribalinae has hitherto been recorded from Lebanon, but it was recorded from a geographically close country, Cyprus (two species; Lackner et al. 2015). Unidentified *Tribalus* occur also in neighboring Syria and Israel (Lackner, unpublished). Thus, the occurrence of *Tribalus* in Lebanon is to be expected.

This study is aimed to investigate the diversity of the Histeridae in different Lebanese regions relating to their colonization of ephemeral resources (carrion and dung). We likewise comment on Histeridae that were collected during field trips that are not necessarily associated to the ephemeral resources. A checklist of species, as well as key to all Lebanese Histeridae (including images of all species) are provided.

## Materials and methods

The majority of specimens were collected in pitfall traps (28 cm height and 16 cm width) baited with rotting fish and pig dung. Specimens were collected after one week of placing the pitfall trap in each locality. The localities and their coordinates are mentioned in Table 1. Collection of specimens was also done during field trips in Baissour, Rechmaya, and Tyre. In Baissour and Rechmaya, the specimens were collected from under stones on the riverside and through sifting soil detritus. In Tyre samples were collected through sand cascading on the beach. Quantitative data on *Atholus*, *Margarinotus*, and *Hypocacculus* were recorded from sampling pitfall traps baited by pig carcasses.

**Table 1.** Sampling localities coordinates and altitude.

District	Locality	Latitude / Longitude	Altitude (m a.s.l.)
**Hasbaya**	Hasbaya	33°23'52.3"N, 35°41.6’6.6"E	750
	Kfeir	33°25'48.0"N, 35°44'22.8"E	909
	Khalwat El Kfeir	33°25'4.6"N, 35°42'59.2"E	1000
	Mimes	33°25'12.0"N, 35°42'59.2"E	789
**Matn**	Fanar	33°52'44"N, 35°34'04"E	250
	Naas-Bikfaya	33°54'42.4"N, 35°40'32.7"E	1090
**Rashaya**	Ain Harcha	33°27'35.2"N, 35°46'45.6"E	994
	Tanoura	33°28'29.1"N, 35°47'58.9"E	985
	Bakifa	33°29'36.5"N, 35°49'8.9"E	994
	Rashaya	33°26'55.7"N, 35°48'58.9"E	1223
	Kfar Qouq	33°32'5.7"N, 35°51'32.6"E	1100
**Shouf-Aley**	Badghan	33°46'4.5"N, 35°40'11.4"E	1211
	Baissour	33°45’32.9"N, 35°34'1.8"E	850
	Misherfeh	33°45'31.5"N, 35°39'17.9"E	950
	Nabaa Al Safa	33°44'58.7"N, 35°41'41.2"E	959
	Rechmaya	33°44'13.2"N, 35°35'56.7"E	450
	Sawfar	33°48'7.9"N, 35°42'8.4"E	1194
**Tyre**	Tyre	33°16'19.2"N, 35°12'12.5"E	0

General observations and dissections were carried out using stereomicroscope Nikon SMZ1500. Without genital extraction, males of *Saprinus* species can be usually recognized through the examination of the anterior tarsal setae, which are expanded and lamellate, whereas they are unexpanded and pointed in female. Often the males possess a longitudinal depression on the metaventrite and occasionally also a single or two tiny tubercles on the apical metaventral margin. Male genitalia were first macerated in 10% KOH solution for ca. 3 hours, cleared in 80% ethanol, macerated in lactic acid with fuchsine, incubated at 60 °C for another 30 min, subsequently cleared in 80 % ethanol, and then observed in α-terpineol in a small dish. Digital photographs of male genitalia were taken by a Nikon 4500 Coolpix camera and edited in Adobe Photoshop CS5. Genitalia drawings based on the photographs or direct observations were produced with the aid of Hakuba klv7000 light box. Habitus photographs were taken by F. Slamka (Bratislava, Slovakia). Specimens were measured with an ocular micrometer. Higher taxa in our paper are arranged according to Mazur (2011); species within higher taxa are aligned alphabetically. For the morphological terminology the reader is referred to Ôhara (1994) and especially Lackner (2010). The general distribution of Histeridae in the Middle East is extrapolated from Lackner et al. (2015). Specimens were identified using the key of Kryzhanovskij and Reichardt (1976) as well as comparing them with reliably identified voucher specimens deposited in the collection of T. Lackner.

The maps of species distribution were made using Google maps and Microsoft Visual Studio Code (Version 1.37).

## Results

List of species recorded from Lebanon; their distribution in the Middle East and biology are mentioned. The list records are based on the Palaearctic catalogue (Lackner et al. 2015) and on our sampling efforts from ephemeral resources, viz. pig carrion, pig dung, and other manual collecting. The species distributions across Lebanese localities are presented on geographic maps. In addition, the checklist, a key, and images of all Histeridae species of Lebanon are provided.

### Histeridae Gyllenhal, 1808 of Lebanon

#### Subfamily Abraeinae W.S. Macleay, 1819

**Distribution.** The subfamily contains five tribes and is distributed worldwide (Mazur 2011). In Lebanon, so far only one species of the tribe Teretriini Bickhardt, 1914 has been recorded.

**Biology.** Members of the Abraeinae subfamily are often found under bark, in rotting wood, inside galleries of xylophagous insects; in the case of Acritini it is decaying vegetable matter that they frequent the most (Kryzhanovskij and Reichardt 1976).

#### Tribe Teretriini Bickhardt, 1914

***Stenopleurum* J. Müller, 1937**

***Stenopleurum rothi* (Rosenhauer, 1856)**

Figure 18

**Distribution in the Middle East.** Cyprus, Lebanon, Syria, Turkey (Lackner et al. 2015).

**Biology.** This species occurs under the bark of coniferous trees, especially pines, where it presumably preys upon larvae of xylophagous insects (Kryzhanovskij and Reichardt 1976).

#### Subfamily Dendrophilinae Reitter, 1909

**Distribution.** This subfamily contains four tribes and is distributed worldwide (Mazur 2011). In Lebanon, so far only two representatives of the tribe Bacaniini Kryzhanovskij, 1976 have been recorded.

**Biology.** The biology of Dendrophilinae is similar to that of Abraeinae, with most taxa being true dendrophiles and several taxa occurring on dung of herbivore mammals (e.g., *Xestipyge* Marseul, 1862).

#### Tribe Bacaniini Kryzhanovskij, 1976

***Abraeomorphus* Reitter, 1886**

**Distribution.** Oriental, Australasian, and Palearctic regions (Mazur 2011).

**Biology.***Abraeomorphus* species occur in rotting wood, often under bark (Kryzhanovskij and Reichardt 1976).

***Abraeomorphus besucheti* Mazur, 1977**

Figure 16

**Distribution in the Middle East.** Israel, Lebanon (Lackner et al. 2015).

**Biology.** As with the general biology of the genus.

***Abraeomorphus minutissimus* (Reitter, 1884)**

Figure 17

**Distribution in the Middle East.** Lebanon (Lackner et al. 2015).

**Biology.** This species is found under bark of oaks (Kryzhanovskij and Reichardt 1976).

#### Subfamily Tribalinae Bickhardt, 1914

**Distribution.** Worldwide (Mazur 2011).

**Biology.** Members of the subfamily are most-commonly found across humid and warm lowland forests, but several taxa are also encountered along streams or rivers. These beetles are often hidden in decaying vegetable debris, but can also be collected from under bark (Kovarik and Caterino 2016; T. Lackner, pers. obs.). According to P.W. Kovarik (pers. comm. 2019) most of the Tribalinae are associated with rotting tree trunks or leaf litter where the larvae prey on soft-bodied insects and adults feed on fungal spores as well as soft-bodied insects.

#### *Tribalus* Erichson, 1834

**Tribalus (Tribalus) sp.**

Figures 3, 120

**Distribution.***Tribalus* contains 65 described species divided into two subgenera and is considered a species-rich genus (Lackner and Vienna 2017). The bulk of its representatives occur in Africa, while a smaller number of taxa are present in the Palaearctic and Oriental regions (Mazur 2011). We collected a presumably undescribed species, which represents the first occurrence of this genus for Lebanon, in Baissour, Fanar, and Rechmaya (Figs 120, 126).

**Biology.** Members of *Tribalus* are found mostly under stones in wetter areas near streams. They can be occasionally collected by sifting forest detritus as well (T. Lackner, pers. obs.). We collected 20 specimens of an unidentified species of *Tribalus* from under stones and tree bark, respectively, in wet areas near rivers of Baissour (8 specimens) and Rechmaya (12 specimens).

#### Subfamily Histerinae Gyllenhal, 1808

**Distribution.** Worldwide (Mazur 2011). The subfamily comprises five tribes: Exosternini Bickhardt, 1914; Histerini Gyllenhal, 1808; Hololeptini Hope, 1840; Omalodini Kryzhanovskij, 1972 and Platysomatini Bickhardt, 1914. The tribe Omalodini is almost exclusively Neotropical and no member of the otherwise widely distributed Hololeptini has been recorded from Lebanon hitherto. Regarding the Platysomatini, only the subcortical species Platylister (Popinus) simeani (Mulsant & Godart, 1875) and Platysoma (Cylister) cornix Marseul, 1861 have yet been recorded from Lebanon. On the other hand, likewise subcortical species Platysoma (Platysoma) compressum (Herbst, 1783) and P. (P.) inexpectatum Lackner, 2004 have been recorded from neighboring Syria; their occurrence in Lebanon therefore cannot be ruled out. As mentioned in the introduction, a single member of the otherwise oriental genus *Notodoma* Lacordaire, 1854, *N. lewisi* Reitter, 1910 has been recorded from Lebanon; this species is otherwise also known from Turkey (Lackner and Hlaváč 2002). A strictly myrmecophilous species *Spathochus coyei* Marseul, 1864 is known from neighboring Syria and Israel, as well as Cyprus or Turkey (Lackner 2009), making its occurrence in Lebanon highly likely. We therefore decided to depict this highly charismatic ant inquiline here as well as include it in the key. The tribe Histerini is the most-widely distributed and most species-rich tribe of the subfamily worldwide.

**Biology.** Members of Histerini are most often encountered on decomposing organic matter, such as manure, dung, compost heaps, decaying vegetables, but are also found on carrion and rotting mushrooms. Inquilinous members are also rather numerous in the subfamily, especially in the Palaearctic, Nearctic and Neotropical regions (Kryzhanovskij and Reichardt 1976). Platysomatini are subcortical as a rule, while Exosternini have varied habits and include fungivores, inquilines and dendrophiles alike (Kovarik and Caterino 2016).

#### Tribe Exosternini Bickhardt, 1914

***Notodoma* Lacordaire, 1854**

**Distribution.***Notodoma* is distributed predominantly in the Oriental region, with a single Palaearctic species, occurring in Lebanon, Syria and Turkey (Mazur 2011).

**Biology.** Mostly found in and on rotting mushrooms where it preys on Diptera that develop on rotting fungi and basidiomycete mushrooms (Kovarik and Caterino 2016).

***Notodoma lewisi* Reitter, 1910**

Figure 5

**Distribution in the Middle East.** Lebanon, Syria, Turkey (Lackner et al. 2015). Described from “Hochsyrien, bei Akbes” (Reitter 1910). This locality (Akbès = Meydan Ekbaz) probably does not lie in Lebanon, but in Turkey, right on the Syrian-Turkish border, between the Turkish town Osmaniye and Syrian town of Aleppo. Lackner and Hlaváč (2002) reported a specimen from south-eastern Turkey (Arslanlı, near Erdemli; misspelled as “Arsanli” in their publication) – their locality is actually quite close to the type locality of this species. Most likely, *Notodoma lewisi* does not occur in Lebanon, but since it has been included in all major catalogues of Histeridae (e.g., Mazur 2011) as described from Lebanon, we decided to keep it here pending further investigation.

**Biology.** A fungivorous, extremely rare species (Lackner and Hlaváč 2002).

#### Tribe Histerini Gyllenhal, 1808

***Atholus* Thomson, 1859**

**Distribution.** The genus *Atholus* comprises 78 species that inhabit Holarctic, Afrotropical and Oriental regions (Mazur 2011).

**Biology.** Members of *Atholus* can be found in decomposing carrion and dung, but are commonly found also under stones and in animal burrows (Penati 2009). Members among this genus can be attracted to *Euphorbia* in xeric areas and to rotting roots of Apiaceae and Fabaceae (Kovarik and Caterino 2016).

***Atholus duodecimstriatus duodecimstriatus* (Schrank, 1781)**

Figures 9, 121

**Distribution in the Middle East.** Iran, Israel, Saudi Arabia, Syria, Turkey (Lackner et al. 2015). Previously reported from Lebanon from Badghan (Shayya et al. 2018) (Fig. 121).

**Biology.** This species shows a preference for dung that has lost much of its moisture; it has likewise been found in association with various stored products where it likely preys on beetle larvae feeding on these materials (Bajerlein 2009; Kovarik and Caterino 2016; Mazur et al. 2017). In Lebanon, *A. duodecimstriatus duodecimstriatus* was attracted to carrion (Shayya et al. 2018). A very common species in Lebanon.

***Eudiplister* Reitter, 1909**

***Eudiplister castaneus* (Ménétriés, 1832)**

Figure 10

**Distribution in the Middle East.** Cyprus, Iran, Iraq, Israel, Jordan, Lebanon, Syria, Turkey (Lackner et al. 2015).

**Biology.** Unknown. Its congeners *Eudiplister peyroni* (Marseul, 1857) and *Eudiplister planulus* (Ménétriés, 1849) were found under plant remains, under stones, under dry excrements, especially in arid places and semi-deserts (Kryzhanovskij and Reichardt 1976).

***Hister* Linnaeus, 1758**

**Distribution.***Hister* is the most species-rich genus of the family and comprises 195 species; these can be found in all world regions, with the exception of Antarctica (Mazur 2011).

**Biology.***Hister* shows preference for dung, but can also be associated with carrion, while some species feed on dung beetle larvae (Coleoptera: Scarabaeidae) present in dung (Kovarik and Caterino 2016).

***Hister limbatus* Truqui, 1852**

Figure 11

**Distribution in the Middle East.** Lebanon, Syria, Turkey (Lackner et al. 2015).

**Biology.** A poorly known species, its biology is unknown.

***Hister sepulchralis* Erichson, 1834**

Figures 12, 121

**Distribution in the Middle East.** Iran, Jordan, Lebanon, Syria, Turkey (Lackner et al. 2015). Herein it is reported from Lebanon for the first time from Kfar Kouq (Fig. 121, 126).

**Biology.***Hister sepulchralis* occurs most often in cattle dung (Rozner 2010) and, like other *Hister* species, it requires liquid fraction from dung to have disappeared to oviposit (Kovarik and Caterino 2016). We collected a singleton of this species from a pig dung-baited pitfall trap. A sporadic and uncommon species in Lebanon.

***Margarinotus* Marseul, 1853**

**Distribution.***Margarinotus* includes ten subgenera containing 109 species altogether, found predominantly in the Holarctic region; several species are likewise autochthonous to the Oriental region (Mazur 2011).

**Biology.** Taxa grouped in *Margarinotus* are varied in their habitat preferences. Several species are linked to carrion or dung, while others prefer rodent burrows (Caterino 2010; Kovarik and Caterino 2016).

**Margarinotus (Ptomister) brunneus (Fabricius, 1775)**

Figures 14, 121, 126

**Distribution in the Middle East.** Iran, Israel, Turkey (Lackner et al. 2015). Previously found in Lebanon (Shayya et al. 2018). Herein, M. (P.) brunneus is reported from the following Lebanese localities: Fanar, Kfeir, Mimes, Naas, Nabaa Al Safa (Fig. 121).

**Biology.** This species shows a clear preference for carrion (Kovarik and Caterino 2016), and has previously been reported from pig carcasses (Matuszewski et al. 2008) as well as from dung (Rozner 2010; Mazur et al. 2017). In Lebanon M. (P.) brunneus was collected from carrion during spring in Fanar (50 specimens) and Naas (181 specimens). It was also collected during the same season from rotting fish-baited pitfall traps in Kfeir (1 specimen), Mimes (1 specimen), and Nabaa Al Safa (6 specimens). A very common and widespread species in Lebanon.

**Margarinotus (Grammostethus) ruficornis (Grimm, 1852)**

Figures 13, 121

**Distribution in the Middle East.** Israel, Jordan, Syria and Turkey (Lackner et al. 2015). In Lebanon, previously mentioned from Fanar (Shayya et al. 2018) (Fig. 121).

**Biology.** Often found in decaying wood in the company of various Formicidae (*Lasius* spp. and *Formica* spp.); it has likewise been known to occur on excrement (Sanchez and Chittaro 2018). In our study, a singleton of M. (G.) ruficornis was collected from carrion during spring in Fanar.

#### Tribe Platysomatini Bickhardt, 1914

***Platylister* Lewis, 1892**

**Distribution.** Genus *Platylister* contains three subgenera and is distributed predominantly across Afrotropical, Oriental, and Australasian regions, with two species recorded also from circum-mediterranean area (Mazur 2011).

**Biology.** Members of *Platylister* are collected under bark of trees, where they prey on (the larvae of) subcortical insects (Kryzhanovskij and Reichardt 1976).

**Platylister (Popinus) simeani (Mulsant & Godart, 1875)**

Figure 7

**Distribution in the Middle East.** Lebanon, Turkey, United Arab Emirates (Lackner et al. 2015).

**Biology.** Attracted to the rotting roots of *Astragalus* (Fabaceae) (Kovarik and Caterino 2016).

***Platysoma* Leach, 1817**

**Distribution.***Platysoma* contains three subgenera and is spread across the whole world, albeit only a single species is known from South America (Mazur 2011).

**Biology.** Associated with bark of trees, where it preys upon members of subcortical insect communities (Kovarik and Caterino 2016).

**Platysoma (Cylister) cornix Marseul, 1861**

Figure 8

**Distribution in the Middle East.** Cyprus, Israel, Lebanon, Syria, Turkey (Lackner et al. 2015).

**Biology.** Found under bark of pines (Kryzhanovskij and Reichardt 1976).

**Onthophilinae Macleay, 1891**

**Distribution.** Worldwide (Mazur 2011).

**Biology.** Members of Onthophilinae have varied habits. They occur in decaying vegetable matter, on dung, and on rotting mushrooms, but the subfamily likewise contains dendrophilous and nidicolous species (Kovarik and Caterino 2016).

***Onthophilus* Leach, 1817**

**Distribution.***Onthophilus* is predominantly Holarctic in distribution, with several species known also from Central America and a single Australian species (Mazur 2011).

**Biology.** Adults prey on fly eggs (but not larvae) and filter feed on the liquid coating of fresh dung; some are known to prey on Diptera that develop on rotting fungi. Their mouthparts bear modified setae that seem to strain particles from liquid (Kovarik and Caterino 2016).

***Onthophilus bickhardti* Reitter, 1909**

Figure 1

**Distribution in the Middle East.** Israel, Lebanon, Turkey (Lackner et al. 2015).

**Biology.** Biology of this rare species is unknown, but most specimens have been collected during November by pitfall traps in higher elevations in Lebanon (Lackner, unpublished).

**Figures 1–5.**
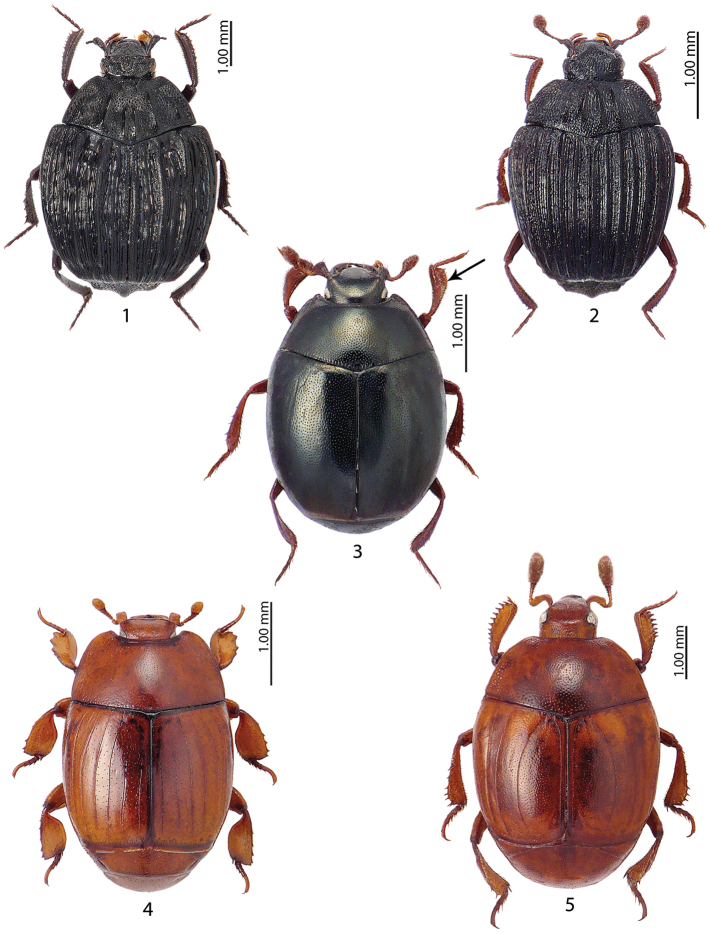
**1***Onthophilus bickhardti* Reitter, 1909 **2***Onthophilus striatus inconditus* Reichardt, 1941 **3***Tribalus* spec. **4***Spathochus coyei* Marseul, 1864 **5***Notodoma lewisi* Reitter, 1910.

***Onthophilus striatus inconditus* Reichardt, 1941**

Figure 2

**Distribution in the Middle East.** Cyprus, Israel, Jordan, Lebanon, Syria, Turkey (Lackner et al. 2015).

**Biology.** Most commonly encountered under decomposing vegetable matter, at times also on outflowing tree sap, in desiccating manure, under carrion or rotting mushrooms (Kryzhanovskij and Reichardt 1976).

**Saprininae C.É. Blanchard, 1845**

**Distribution.** Worldwide (Mazur 2011).

**Biology.**Saprininae have witnessed a remarkable ecological evolution. They are known as colonizers of different ecological niches: ant-nests, dead termitaria, rodent burrows etc. They even gained fine morphological adaptations and distribution throughout Old World deserts. In addition, members of Saprininae have colonized mammal burrows, nests of birds, ants, termites, and even tortoise burrows. Their life histories are varied, as several lineages exhibit diversity in their terrestrial niches (Lackner 2014a).

***Chalcionellus* Reichardt, 1932**

**Distribution.** Palearctic, Oriental, and Afrotropical regions; a single Afrotropical species has been introduced into Australia (Mazur 2011).

**Biology.** Members of *Chalcionellus* are found in manure, in excrements and on carcasses; the genus contains also a single species occupying the rhizosphere of plants (Kryzhanovskij and Reichardt 1976).

***Chalcionellus blanchii blanchii* (Marseul, 1855)**

Figure 20

**Distribution in the Middle East.** Iran, Iraq, Israel, Lebanon, Syria, Turkey (Lackner et al. 2015).

**Biology.** Like its congeners, found on carcasses and excrements (Kryzhanovskij and Reichardt 1976; T. Lackner, pers. obs.).

***Chalcionellus libanicola* (Marseul, 1870)**

Figure 21

**Distribution in the Middle East.** Lebanon, Syria, Turkey (Lackner et al. 2015).

**Biology.** Unknown, a rare taxon (Lackner 2011).

***Chalcionellus aemulus* (Illiger, 1807)**

Figure 19

**Distribution in the Middle East.** Iran, Israel, Jordan, Lebanon, Turkey (Lackner et al. 2015).

**Biology.** Found on carcasses, in excrements etc. (T. Lackner pers. obs.).

***Gnathoncus* Jacquelin du Val, 1857**

**Distribution.** Predominantly Holarctic in distribution; a single species is known from tropical Africa (Mazur 2011). From the Oriental region a handful of cave-dwelling species have been recorded (Lackner 2020).

**Biology.** Members of *Gnathoncus* occur predominantly in bird nests or burrows of smaller mammals; occasionally they are found also on carrion or decomposing vegetable matter (Kryzhanovskij and Reichardt 1976).

**Figures 6–12.**
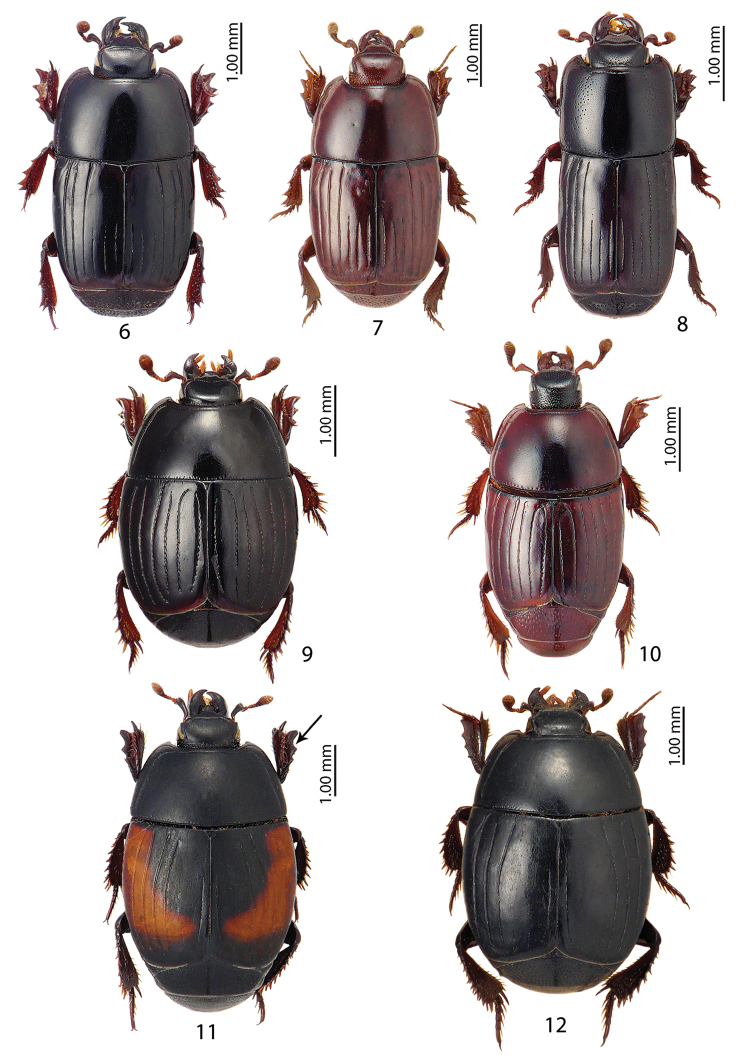
**6**Platylister (Popinus) algiricus (Lucas, 1846) **7**Platylister (Popinus) simeani (Mulsant & Godart, 1875) **8**Platysoma (Cylister) cornix Marseul, 1861 **9***Atholus duodecimstriatus duodecimstriatus* (Schrank, 1781) **10***Eudiplister castaneus* (Ménétriés, 1832) **11***Hister limbatus* Truqui, 1852 **12***Hister sepulchralis* Erichson, 1834.

***Gnathoncus disjunctus suturifer* Reitter, 1896**

Figure 22

**Distribution in the Middle East.** Lebanon, Syria, Turkey (Lackner et al. 2015).

**Biology.** This species is present in burrows of small rodents, e.g., *Citellus* sp. (Kryzhanovskij and Reichardt 1976).

***Hemisaprinus* Kryzhanovskij, 1976**

**Distribution.***Hemisaprinus* contains three described Palaearctic species; one species (*H. subvirescens*) marginally enters also the Oriental region (Mazur 2011).

**Biology.** Members of *Hemisaprinus* are usually associated with carcasses or decomposing vegetable matter (Lackner 2014b).

***Hemisaprinus subvirescens* (Ménétriés, 1832)**

Figures 23, 122, 126

**Distribution in the Middle East.** Cyprus, Iran, Iraq, Israel, Jordan, Syria, Turkey (Lackner et al. 2015). Newly reported from Lebanon (Bakifa) (Fig. 122).

**Biology.** Found chiefly on carrion in arid regions (Reichardt 1941; Lackner 2014b); a forensically relevant species (Su et al. 2013). One specimen was found on decomposing fish-baited pitfall trap.

***Hypocacculus* Bickhardt, 1914**

**Distribution.** Genus *Hypocacculus* contains three subgenera and includes 21 described species, distributed mostly in the Palaearctic and Afrotropical regions (Mazur 2011).

**Biology.** Taxa included in *Hypocacculus* are typically collected from carrion and animal excrement and usually found in dry and arid regions. Also, they can be collected in open landscapes and some are psammophiles (Lackner 2010).

**Hypocacculus (Hypocacculus) metallescens (Erichson, 1834)**

Figures 25, 122, 126

**Distribution in the Middle East.** Cyprus, Israel, Iran, Iraq, Oman, Saudi Arabia, Syria (Lackner et al. 2015). Already mentioned from Lebanon from Hasbaya (Shayya et al. 2018) (Fig. 122).

**Biology.** This species is found in association with small animal carcasses, excrements and other decomposing matter. It also found on coastal dunes in the rhizosphere of psammophilous Graminaceae (Penati 2009). It is a generalist predator (Lackner 2014a). Already reported from Lebanon (Shayya et al. 2018).

**Hypocacculus (Colpellus) praecox (Erichson, 1834)**

Figure 24

**Distribution in the Middle East.** Cyprus, Iran, Israel, Lebanon, Oman, Saudi Arabia, Syria, Turkey, United Arab Emirates, Yemen (Lackner et al. 2015).

**Biology.**Hypocacculus (C.) praecox is a psammo-halobiotic species, which frequents coastal dunes near the roots of halophilous plants and can be attracted to animal carcasses (Penati 2009).

***Hypocaccus* C.G. Thomson, 1867**

**Distribution.***Hypocaccus* contains three subgenera: *Hypocaccus* s. str., *Baeckmanniolus* Reichardt, 1926 and *Nessus* Reichardt, 1932 and its members are distributed almost across the whole world, being poorly represented in South America and Australasia (Mazur 2011).

**Biology.** Members of *Hypocaccus* s. str. and *Baeckmanniolus* are coastal wrack specialists, occasionally occurring also on banks of rivers and lakes, while members of the subgenus Nessus are typical generalist predators with several psammophile or inquiline forms (Kryzhanovskij and Reichardt 1976).

**Figures 13–18.**
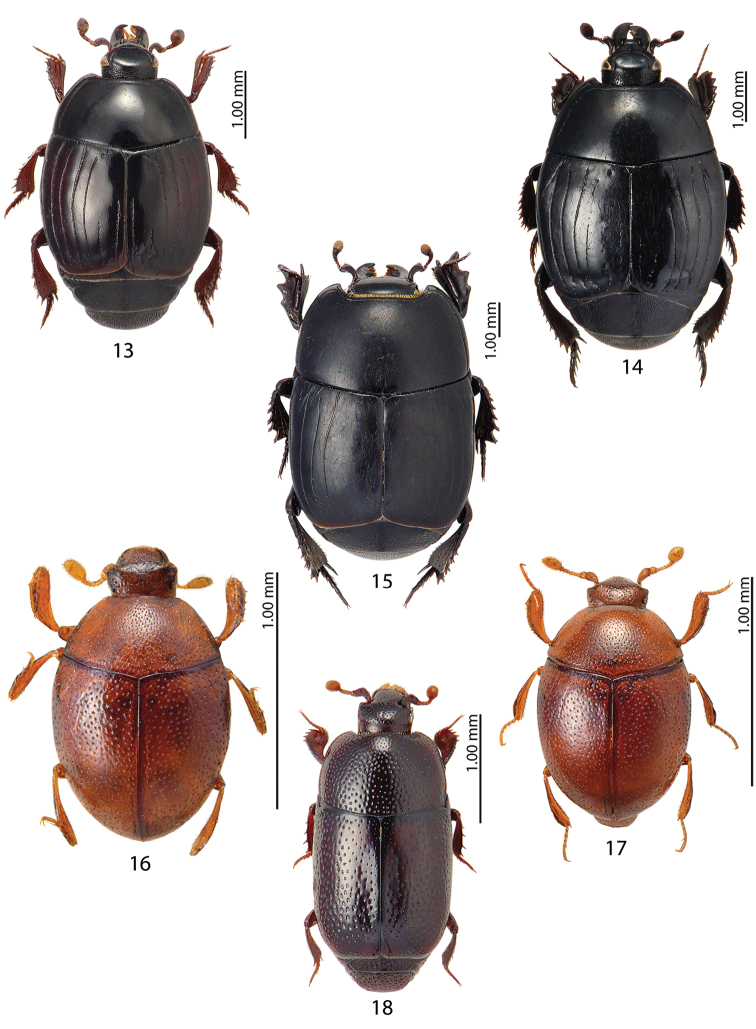
**13**Margarinotus (Grammostethus) ruficornis (Grimm, 1852) **14**Margarinotus (Ptomister) brunneus (Fabricius, 1775) **15**Margarinotus (Stenister) graecus graecus (Brullé, 1832) **16***Abraeomorphus besucheti* Mazur, 1977 **17***Abraeomorphus minutissimus* (Reitter, 1884) **18***Stenopleurum rothi* (Rosenhauer, 1856).

**Hypocaccus (Nessus) baudii (J. Schmidt, 1890)**

Figure 26

**Distribution in the Middle East.** Cyprus, Israel, Lebanon, Syria (Lackner et al. 2015).

**Biology.** Virtually unknown; a rare species.

***Saprinus* Erichson, 1834**

**Distribution.***Saprinus* includes two subgenera *Phaonius* Reichardt, 1941 and *Saprinus* s.str. and 157 species distributed around the world (Mazur 2011). With 116 species in the Palaearctic region and 14 species in Lebanon, it is the most species-rich genus of the Saprininae (Lackner 2010, Lackner et al. 2015, Shayya et al. 2018). Moreover, this study adds three new species records of this genus for the Lebanese fauna. Most of the *Saprinus* species occur in the Palaearctic and Afrotropical regions (Lackner 2010).

**Biology.***Saprinus* shows preference to open xeric landscapes and only few are known from mesic biotopes (Lackner 2010). They are frequent on carrion and less so on dung, and prey on larvae and eggs of soft-bodied insects; in some cases they can capture adult flies on dung (Carlton et al. 1996). Some species could also be found on flowers (Lackner 2010; Kovarik and Caterino 2016).

**Saprinus (Saprinus) aegialius Reitter, 1884**

**Distribution in the Middle East.** Iran, Lebanon, Syria, Turkey (Lackner et al. 2015).

**Biology.** This species is present on carcasses, in excrements, manure, mammal burrows and occasionally even on flowers (Kryzhanovskij and Reichardt 1976).

**Saprinus (Saprinus) calatravensis Fuente, 1899**

Figures 29, 122, 126

**Distribution in the Middle East.** Iran, Israel, Oman, Saudi Arabia, Turkey (Lackner et al. 2015). Already reported from Lebanon (Deir El-Ahmar, Hasbaya; Shayya et al. 2018) (Fig. 122).

**Biology.** An essentially necrophilous taxon, attracted to small- and medium-sized carrion (Faria e Silva et al. 2006; Shayya et al. 2018) frequenting xeric landscapes and sandy soils (Kryzhanovskij and Reichardt 1976) with preference to habitats at lower elevations and restricted to mesomediterranean holm oak (*Quercus ilex* L.) forests in Spain (Martín-Vega et al. 2015). We report three specimens collected from rotting fish-baited pitfall trap in Kfar Qouq.

**Figures 19–26.**
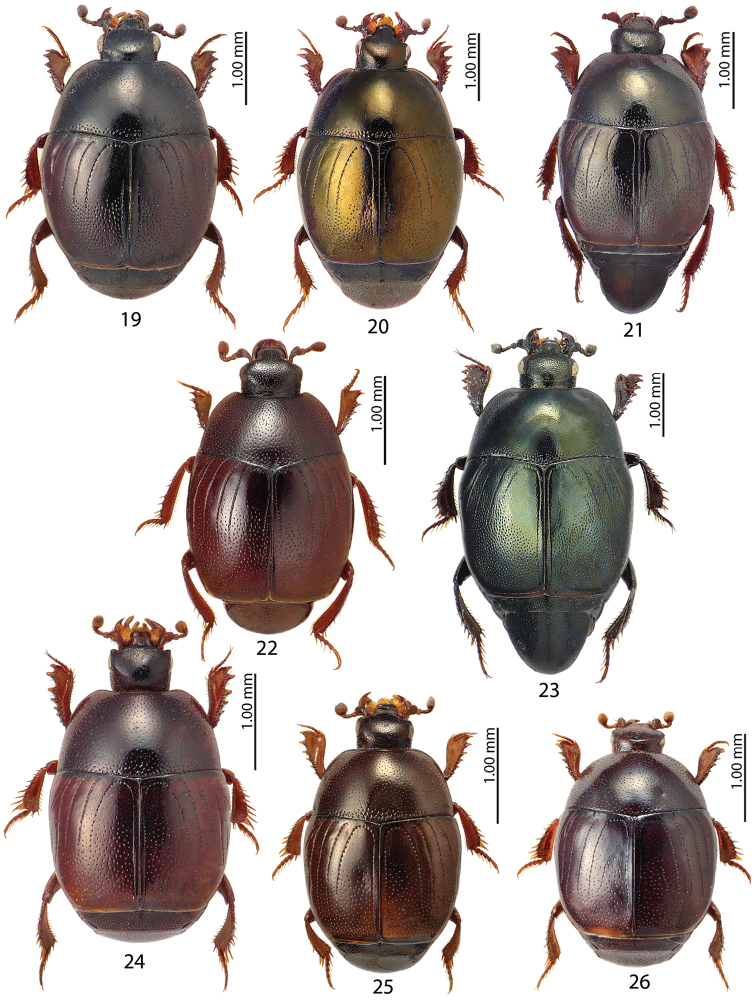
**19***Chalcionellus aemulus* (Illiger, 1807) **20***Chalcionellus blanchii blanchii* (Marseul, 1855) **21***Chalcionellus libanicola* (Marseul, 1870) **22***Gnathoncus disjunctus suturifer* Reitter, 1896 **23***Hemisaprinus subvirescens* (Ménétriés, 1832) **24**Hypocacculus (Colpellus) praecox (Erichson, 1834) **25**Hypocacculus (Hypocacculus) metallescens (Erichson, 1834) **26**Hypocaccus (Nessus) baudii (J. Schmidt, 1890).

**Saprinus (Saprinus) chalcites (Illiger, 1807)**

Figures 30, 122, 126

**Distribution in the Middle East.** Cyprus, Iran, Iraq, Israel, Jordan, Kuwait, Oman, Saudi Arabia, Syria, Turkey, Yemen (Lackner et al. 2015). Already reported from pig carrion in Lebanon (Hasbaya, Fanar, Badghan, Deir El-Ahmar, Naas; Shayya et al. 2018). In our current study it was collected from Badghan, Kfar Qouq, Kfeir, Misherfeh, Nabaa Al Safa and Tanoura (Fig. 122).

**Biology.** A typical saprobiont, attracted to carrion and mammal dung (Lackner and Vienna 2017), found also on rotting vegetable substances. We found it in rotting fish-baited pitfall traps during spring in Badghan (5 specimens), Misherfeh (1 specimen), Nabaa Al Safa (1 specimen), and Tanoura (6 specimens). We likewise collected this species from pig dung-baited pitfall traps during the same season in Kfar Qouq (1 specimen) and Kfeir (1 specimen).

**Saprinus (Saprinus) externus (Fischer von Waldheim, 1823)**

Figures 31, 123, 126

**Distribution in the Middle East.** Iran, Jordan, Syria, Turkey (Lackner et al. 2015). Newly reported from Lebanon (Kfar Qouq and Rashaya).

**Biology.** Found among carrion entomofauna at various stages of decomposition, especially in rural areas (Al Tunsoy et al. 2017). Saprinus (S.) externus is an infrequent taxon in Lebanon. According to Reichardt (1941), this species is linked to carrion and dung. In our samplings, a singleton was reported from pig dung-baited pitfall trap in Kfar Kouq and another was collected in decomposing fish-baited pitfall trap in Rashaya (Fig. 123).

**Figures 27–33.**
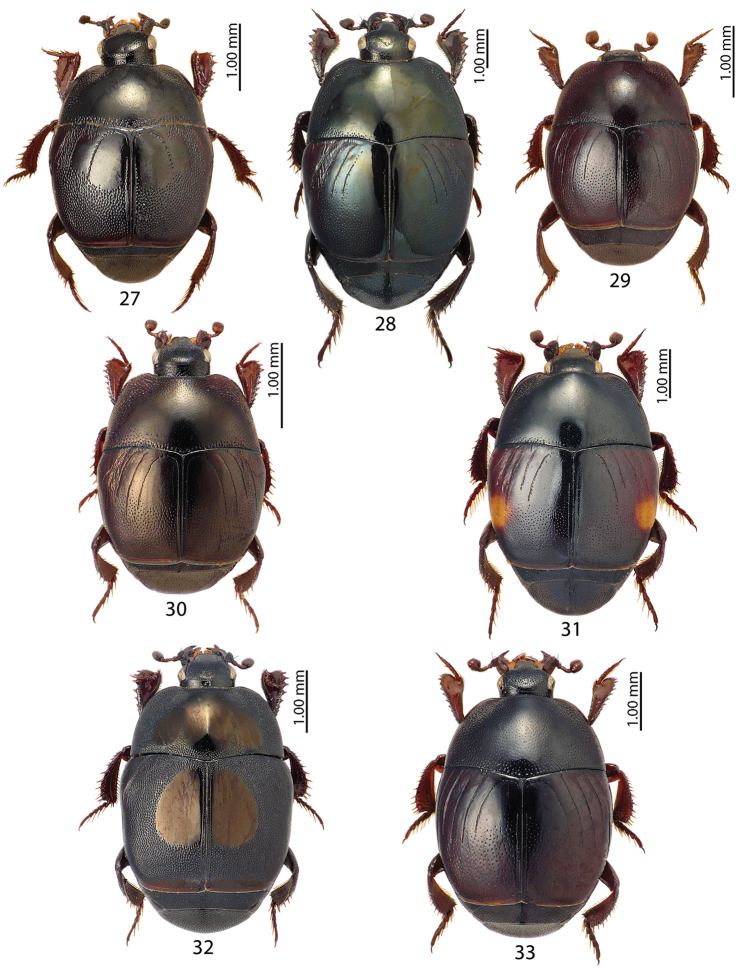
**27**Saprinus (Saprinus) aegialius Reitter, 1884, **28**Saprinus (Saprinus) caerulescens caerulescens (Hoffmann, 1803), **29**Saprinus (Saprinus) calatravensis Fuente, 1899, **30**Saprinus (Saprinus) chalcites (Illiger, 1807), **31**Saprinus (Saprinus) externus (Fischer von Waldheim, 1823), **32**Saprinus (Saprinus) figuratus Marseul, 1855, **33**Saprinus (Saprinus) godet (Brullé, 1832).

**Saprinus (Saprinus) figuratus Marseul, 1855**

Figures 32, 123, 126

**Distribution in the Middle East.** Israel, Jordan, Oman, Saudi Arabia, Syria (Lackner et al. 2015). Newly reported from Lebanon (Ain Harsha and Rashaya).

**Biology.**Saprinus (S.) figuratus occurs on carrion, with restriction to the meso-mediterranean holm oak forests on basic soils (Reichardt 1941; Martín-Vega et al. 2015). Herein, one specimen was found in a decomposing fish pitfall trap in Rashaya, and another specimen was found in a similar trap in Ain Harsha (Fig. 123).

**Saprinus (Saprinus) godet (Brullé, 1832)**

Figures 33, 123, 126

**Distribution in the Middle East.** Turkey, Saudi Arabia (Lackner et al. 2015). Already mentioned from Lebanon (Fanar, Badghan, Deir El-Ahmar, Hasbaya, Sin El-Fil; Shayya et al. 2018). We herein report it from Badghan, Kfeir, Mimes, Misherfeh, Nabaa Al Safa, and Tanoura (Fig. 123).

**Biology.** Occurs on carcasses (Kryzhanovskij and Reichardt 1976; Penati 2009; Shayya et al. 2018). In Lebanon we sampled it from rotting fish-baited pitfall trap during spring in Badghan (1 specimen), Mimes (1 specimen), Misherfeh (2 specimens), Nabaa Al Safa (1 specimen), and Tanoura (1 specimen). In addition, it was sampled from pig dung-baited pitfall traps in Kfeir (2 specimens).

**Saprinus (Saprinus) niger (Motschulsky, 1849)**

Figures 36, 123, 126

**Distribution in the Middle East.** Iran, Iraq, Israel, Jordan, Syria, Turkey (Lackner et al. 2015). New to Lebanon (Kfar Qouq) (Fig. 123).

**Biology.** A member of the carrion entomofauna (Reichardt 1941). We recorded it from a rotting fish-baited pitfall trap during spring in Kfar Qouq (1 specimen).

**Figures 34–39.**
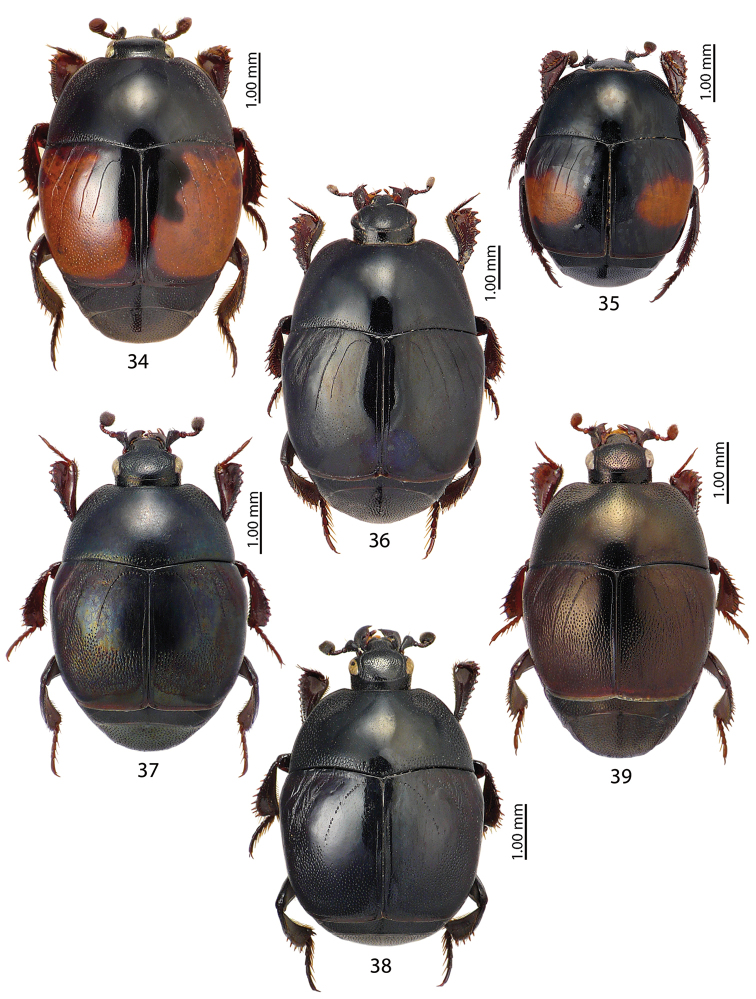
**34**Saprinus (Saprinus) maculatus (P. Rossi, 1792) **35**Saprinus (Saprinus) magnoguttatus J. Müller, 1937 **36**Saprinus (Saprinus) niger Motschulsky, 1849 **37**Saprinus (Saprinus) prasinus prasinus Erichson, 1834 **38**Saprinus (Saprinus) robustus Krása, 1944 **39**Saprinus (Saprinus) strigil Marseul, 1855.

**Saprinus (Saprinus) robustus Krása, 1944**

Figures 38, 124, 126

**Distribution in the Middle East.** Cyprus, Iran, Israel, Jordan, Lebanon, Syria, Turkey (Lackner et al. 2015). Already reported from Lebanon from Deir El-Ahmar and Hasbaya (Shayya et al. 2018). We herein add further Lebanese localities: Ain Harsha, Kfeir, Misherfeh and Sawfar (Fig. 124).

**Biology.**Saprinus (S.) robustus inhabits dung and carrion alike (Anlaş et al. 2007; Shayya et al. 2018). It was examined in a rotting fish-baited pitfall trap during spring in Ain Harsha (2 specimens), Misherfeh (2 specimens), and Sawfar (3 specimens). A single specimen was attracted to pig dung-baited pitfall trap during spring in Kfeir.

**Saprinus (Saprinus) strigil Marseul, 1855**

Figures 39, 124, 126

**Distribution in the Middle East.** Cyprus, Iran, Iraq, Israel, Oman, Saudi Arabia, Syria, Yemen (Lackner et al. 2015). We already reported it from Lebanon (Hasbaya, Badghan, Fanar, Deir El-Ahmar and Naas; Shayya et al. 2018). New Lebanese localities are: Badghan, Kfar Qouq, Kfeir, Khalwat El Kfeir, Mimes, Misherfeh, Nabaa Al Safa, Rashaya, and Tanoura (Fig. 124).

**Biology.**Saprinus (S.) strigil was encountered on carrion (Shayya et al. 2018). We collected it from rotting fish-baited pitfall trap during spring in Badghan (12 specimens), Mimes (8 specimens), Misherfeh (25 specimen), Nabaa Al Safa (9 specimens), Rashaya (1 specimen), and Tanoura (8 specimens). It was also found in a pig dung-baited pitfall traps in Kfar Kouq (3 specimens), Kfeir (1 specimen), and Khalwat El Kfeir (5 specimens).

**Figures 40–43.**
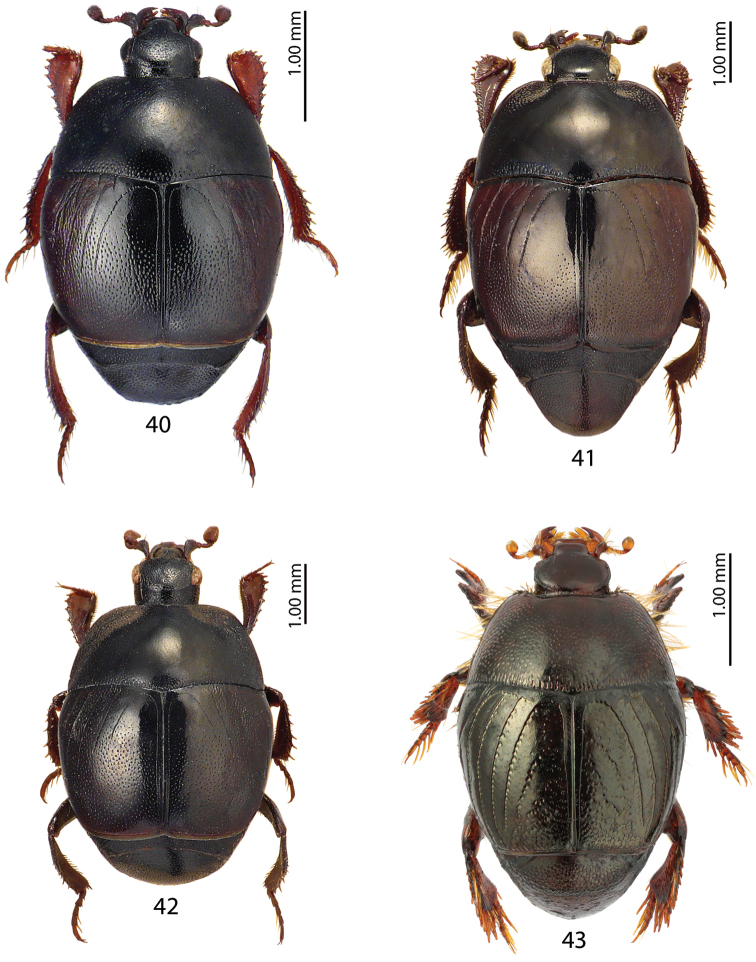
**40**Saprinus (Saprinus) submarginatus J. Sahlberg, 1913 **41**Saprinus (Saprinus) subnitescens Bickhardt, 1909 **42**Saprinus (Saprinus) tenuistrius sparsutus Solsky, 1909 **43***Xenonychus tridens* (Jacquelin du Val, 1853).

**Saprinus (Saprinus) subnitescens Bickhardt, 1909**

Figures 41, 125, 126

**Distribution in the Middle East.** Cyprus, Iran, Iraq, Israel, Lebanon, Syria, Turkey (Lackner et al. 2015). Already known from Lebanon (Fanar, Badghan, Naas, Deir El-Ahmar and Hammana; Shayya et al. 2018). We herein report this species from the following Lebanese localities: Badghan, Bakifa, Kfeir, Mimes, Misherfeh, Nabaa Al Safa, Rashaya, and Sawfar (Fig. 125).

**Biology.**Saprinus (S.) subnitescens is a predator without an obvious habitat preference; it has been found on carrion (Özdemir and Sert 2008; Rozner 2010; Al Tunsoy et al. 2017; Shayya et al. 2018; Martín-Vega et al. 2015), and likewise on manure and decaying vegetable matter (Bousquet and Laplante 2006). We collected it from rotting fish-baited pitfall traps during spring in Badghan (1 specimen), Bakifa (1 specimen), Mimes (1 specimen), Misherfeh (20 specimen), Nabaa Al Safa (49 specimen) and Sawfar (2 specimens). Two specimens were found in a pig dung-baited pitfall trap in Kfeir.

**Saprinus (Saprinus) tenuistrius sparsutus Solsky, 1876**

Figures 42, 125, 126

**Distribution in the Middle East.** Iran, Iraq, Israel, Syria, Turkey (Lackner et al. 2015). According to Rozner (2010) as well as our observations S. (S.) tenuistrius sparsutus is a frequent taxon in the Eastern Mediterranean area. Already known from Lebanon (Deir El-Ahmar, Hasbaya; Shayya et al. 2018). New Lebanese localities: Khalwat El Kfeir, and Rashaya (Fig. 125).

**Biology.** It is known among the entomofauna of carrion (Al-Tunsoy et al. 2017; Shayya et al. 2018). In Spain, it was found in meso-and supra-Mediterranean forests, which is similar to our findings in Lebanon (Martín-Vega et al. 2015). It is noteworthy to mention that Lebanon shares a similar Mediterranean climate with Spain. We collected it from rotting fish-baited pitfall trap in Rashaya (1 specimen) and from pig dung baited pitfall trap in Khalwat El Kfeir (1 specimen).

**Figures 44–56.**
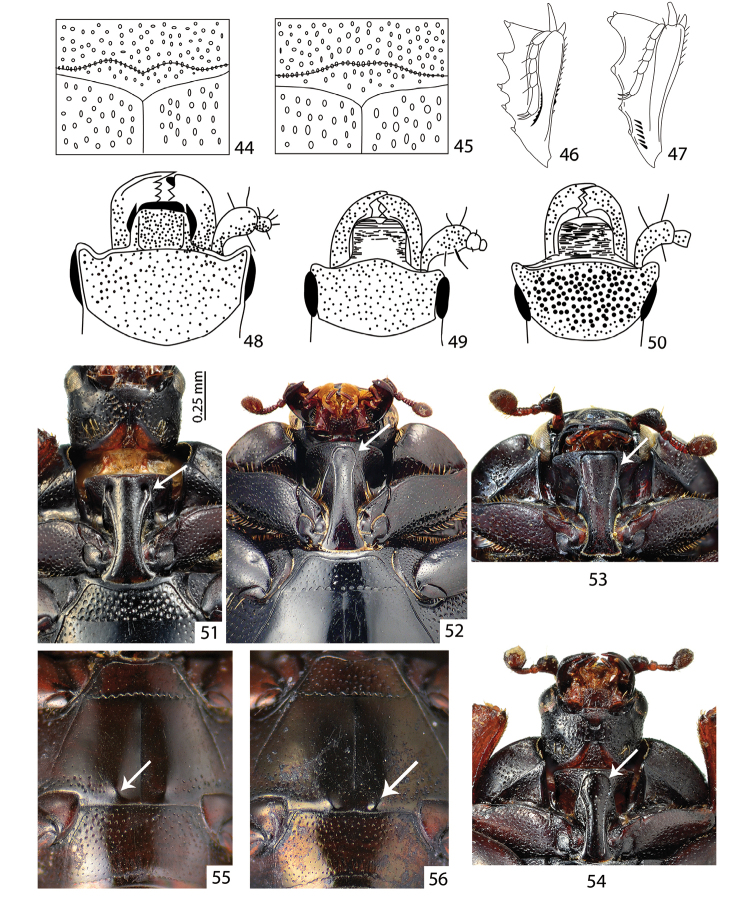
**44***Abraeomorphus besucheti* Mazur, 1977– pronotum (re-drawn from Mazur (1977)) **45***Abraeomorphus minutissimus* (Reitter, 1884) – pronotum (re-drawn from Mazur (1977)) **46**Platysomatini, protibia (re-drawn from Kryzhanovskij and Reichardt (1976)) **47**Histerini, protibia (re-drawn from Kryzhanovskij and Reichardt (1976)) **48**Hypocacculus (Colpellus) praecox (Erichson, 1834) – frons (re-drawn from Kryzhanovskij and Reichardt (1976)) **49**Hypocacculus (Hypocacculus) metallescens (Erichson, 1834) – frons (re-drawn from Kryzhanovskij and Reichardt (1976)) **50**Hypocaccus (Nessus) baudii (J. Schmidt, 1890) – frons (re-drawn from Kryzhanovskij and Reichardt (1976)) **51***Hemisaprinus subvirescens* (Ménétriés, 1832) – prosternum **52**Saprinus (Saprinus) niger Motschulsky, 1849 – prosternum **53**Saprinus (Saprinus) subnitescens Bickhardt, 1909 – prosternum **54**Saprinus (Saprinus) submarginatus J. Sahlberg, 1913 – prosternum **55**Saprinus (Saprinus) calatravensis Fuente, 1899 – metaventrite **56**Saprinus (Saprinus) chalcites (Illiger, 1807) – metaventrite.

***Xenonychus* Wollaston, 1864**

**Distribution.***Xenonychus* contains three described species: *Xenonychus tridens* (Jacquelin du Val, 1853) is distributed from the Cape Verde Archipelago and Canary Islands in the west through the Sahara Belt along the Mediterranean coast to the Arabian Peninsula in the east. *Xenonychus aralocaspius* Kryzhanovskij, 1976 is found around the Caspian and Aral Seas, and further inland in the middle Asian countries of Kazakhstan, Uzbekistan and Turkmenistan, while *Xenonychus somaliensis* (Thérond, 1963) is, so far, known exclusively from Somalia (Lackner 2012).

**Biology.** The first two species are inhabitants of arid areas of shifting sand, frequent on sand dunes on beaches and also present inland. The biology of the *X. somaliensis* is unknown, but presumably similar to congeners (Lackner 2012).

***Xenonychus tridens* (Jacquelin du Val, 1853)**

Figures 43, 125

**Distribution in the Middle East.** Cyprus, Israel, Oman, Saudi Arabia, Syria, Turkey, United Arab Emirates (Lackner et al. 2015). New to Lebanon (Tyre) (Fig. 125).

**Biology.** A typical psammo-halobiotic species, usually found under plants on coastal as well as inland dunes; occasionally found also under carrion on sandy surfaces (T. Lackner, pers. obs. 2012). According to Reichardt (1941), it was examined in dune sands of the sea coast at a depth of 15 to 30 cm of a raw layer of sand and in the vicinity of plants roots. During sand-cascading at the Tyre beach, 12 specimens were collected in the rhizosphere of various plants.

**Figures 57–92.**
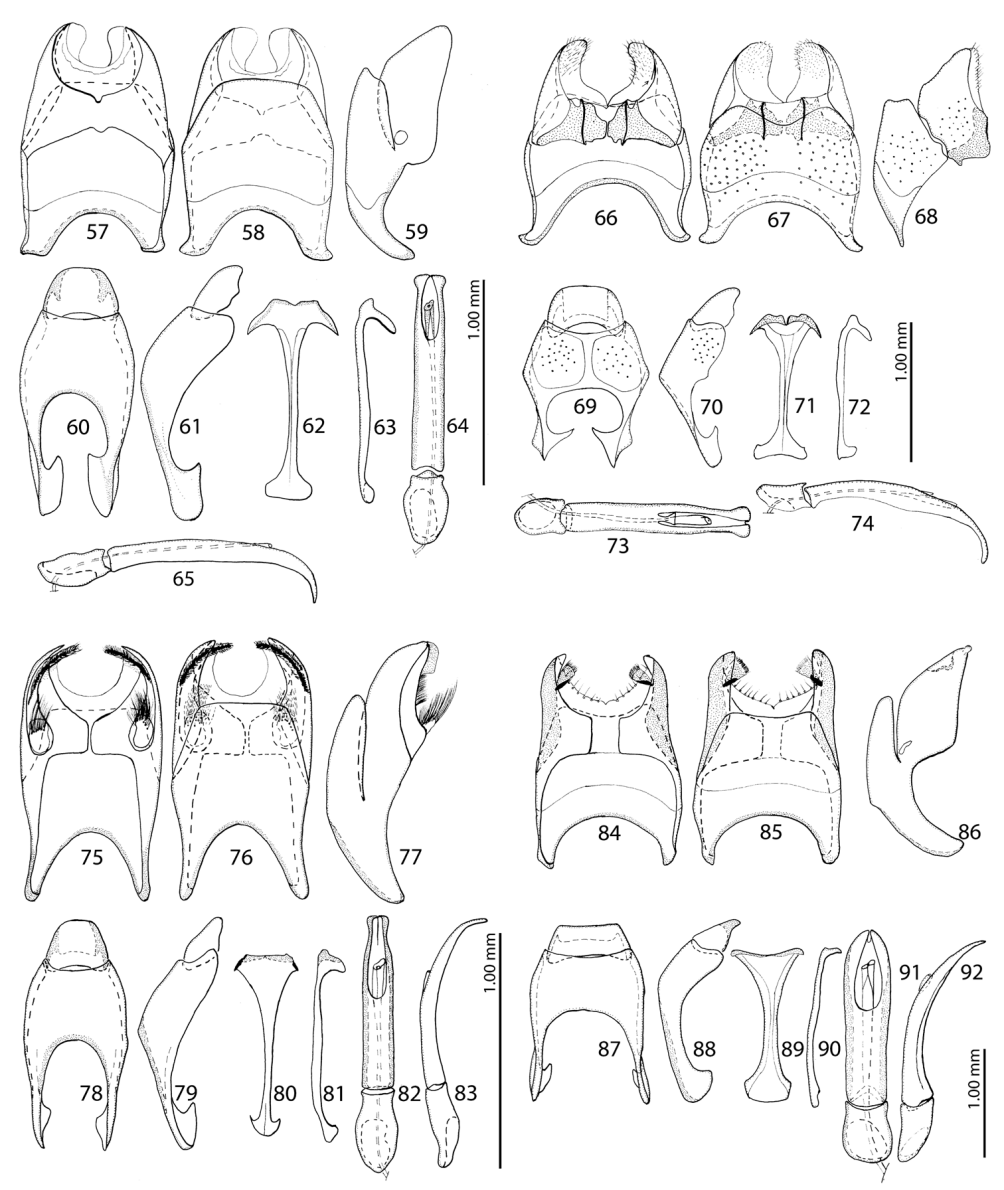
**57–65**Saprinus (Saprinus) subnitescens Bickhardt, 1909 – male genitalia **66–74**Saprinus (Saprinus) robustus Krása, 1944 – male genitalia **75–83**Saprinus (Saprinus) godet (Brullé, 1832) – male genitalia **84–92**Saprinus (Saprinus) tenuistrius sparsutus Solsky, 1909 – male genitalia.

**Figures 93–119.**
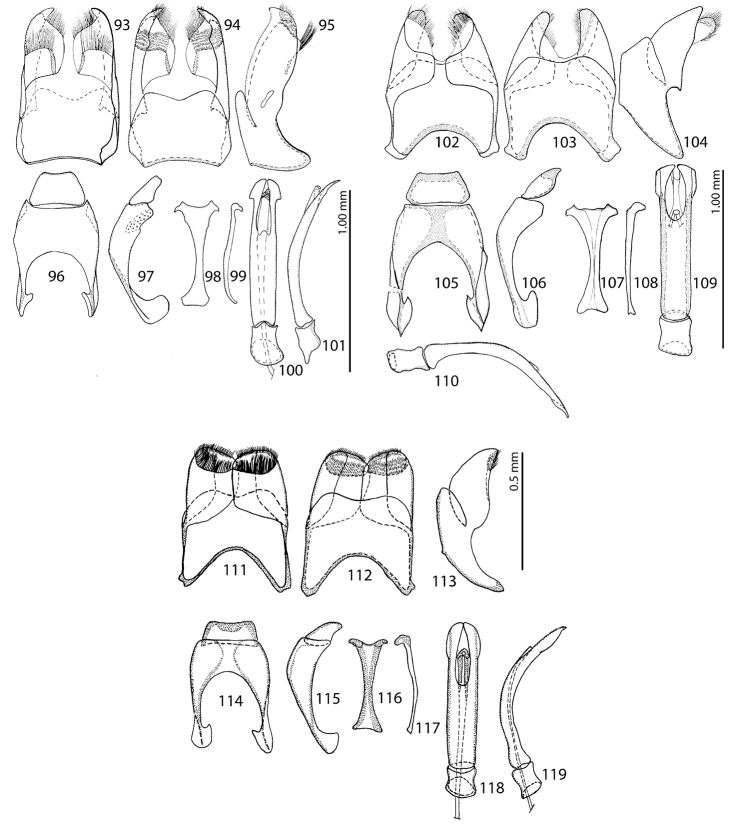
**93–101**Saprinus (Saprinus) submarginatus J. Sahlberg, 1913 – male genitalia **102–110**Saprinus (Saprinus) calatravensis Fuente, 1899 – male genitalia **111–119**Saprinus (Saprinus) chalcites (Illiger, 1807) – male genitalia.

**Figures 120–125.**
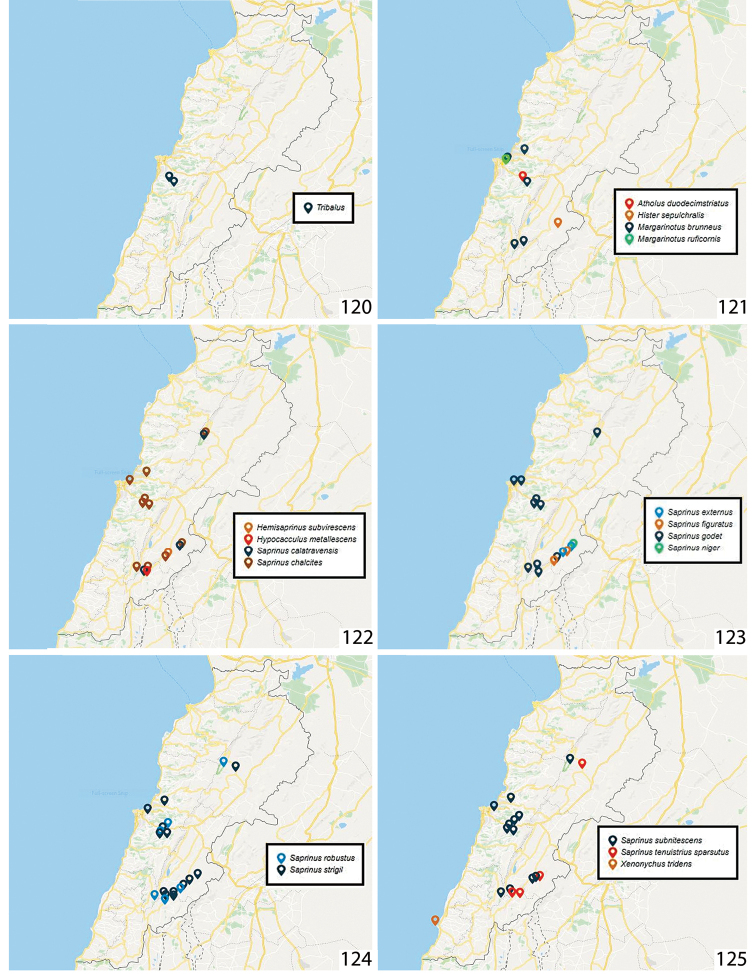
**120** Distribution of *Tribalus* spec. in Lebanon **121** Distribution of *Atholus duodecimstriatus duodecimstriatus*, *Hister sepulchralis*, Margarinotus (Ptomister) brunneus and Margarinotus (Grammostethus) ruficornis in Lebanon **122** Distribution of *Hemisaprinus subvirescens*, Hypocacculus (Hypocacculus) metallescens, Saprinus (Saprinus) calatravensis and Saprinus (Saprinus) chalcites in Lebanon **123** Distribution of Saprinus (Saprinus) externus, Saprinus (Saprinus) figuratus, Saprinus (Saprinus) godet and Saprinus (Saprinus) niger in Lebanon **124** Distribution of Saprinus (Saprinus) robustus and Saprinus (Saprinus) strigil in Lebanon **125** Distribution of Saprinus (Saprinus) subnitescens, Saprinus (Saprinus) tenuistrius sparsutus and *Xenonychus tridens* in Lebanon.

**Figure 126.**
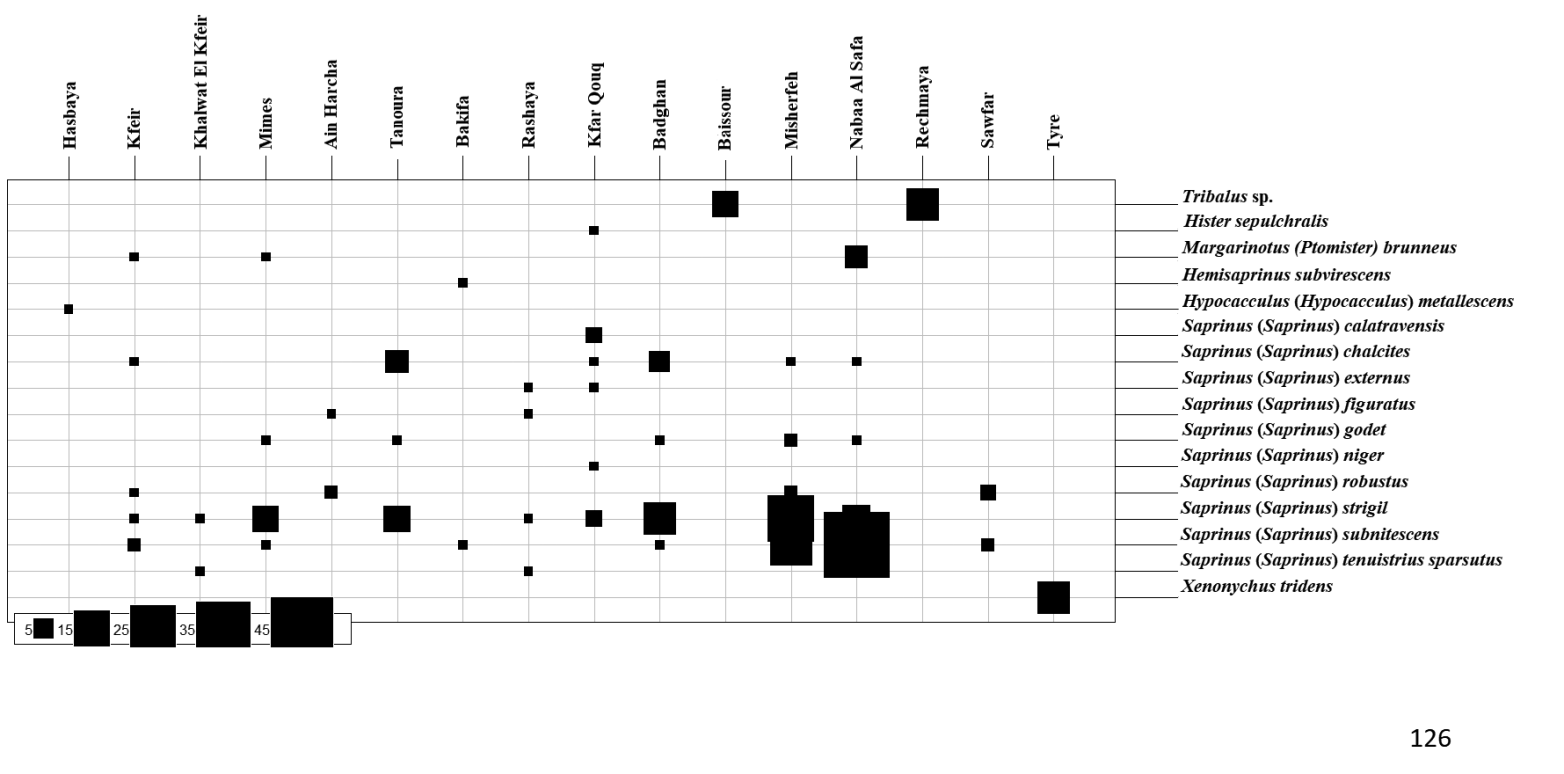
Abundance of Histeridae species collected during this study from different Lebanese localities.

### Key to the Histeridae of Lebanon

We should like to stress that our key contains only species recorded from the territory of Lebanon, with a single exception of *Spathochus coyei*, which has been recorded from the neighboring countries and we strongly suspect it might also occur in Lebanon. If a histerid specimen from Lebanon cannot be identified using our key, we advocate using the monograph of the USSR fauna by Kryzhanovskij and Reichardt (1976; in Russian), which contains keys covering many taxa occurring in the Middle East. Our key will need revision, as the country’s fauna becomes better known.

**Table:** 

1(4)	Taxa of minute size, PEL = max 1.10 mm	***Abraeomorphus* Reitter, 1886**
2(3)	Metaventrite densely punctate, basal pronotal stria medially not distinctly inwardly angulate (Figs 17; 45)	***A. minutissimus* (Reitter, 1884)**
3(2)	Metaventrite sparsely punctate (for fig. see Mazur, 1977 fig. 2); basal pronotal stria medially distinctly inwardly angulate (Figs 16; 44)	***A. besucheti* Mazur, 1977**
4(1)	Larger taxa, PEL > 1.10 mm	**5**
5(8)	Elytra and pronotum with costae	***Onthophilus* Leach, 1817**
6(7)	Large species, PEL = 4.20 mm; punctures of pronotum not forming elongate rugae; pronotum medially with two interrupted keels (Fig. 1)	***O. bickhardti* Reitter, 1909**
7(6)	Smaller species, PEL = max 2.50 mm; punctures of pronotum forming elongate rugae; pronotum medially with four complete keels (Fig. 2)	***O. striatus inconditus* Reichardt, 1941**
8(5)	Elytra and pronotum without costae	**9**
9(34)	Prosternum with prosternal lobe or “presternum” (for fig. see e.g. Ôhara 1994, fig. 11C)	**10**
10(11)	Labrum with setae; protibia with numerous tiny denticles (Figs 3, 120)	***Tribalus* sp.**
11(10)	Labrum asetose; protibia usually with several large teeth topped by denticles, never with numerous tiny denticles (Fig. 11)	**subfamily Histerinae Gyllenhall, 1808**
12(15)	Mesoventrite produced into an anterior angle that fits into an angular emargination of the prosternum (for fig. see e.g. Kanaar 1997, fig. 2)	**tribe Exosternini Bickhardt, 1914**
13(14)	Larger species, PEL > 3.00 mm; body strongly convex; elytral stria IV basally connected with complete sutural elytral stria; antennal club larger than antennal funicle (Fig. 5)	***Notodoma lewisi* Reitter, 1910**
14(13)	Smaller species, PEL < 3.00 mm; body rather flattened; elytral stria IV basally shortened, not connected with shortened sutural elytral stria; antennal club smaller than antennal funicle (Fig. 4)	***Spathochus coyei* Marseul, 1864**
15(13)	Mesoventrite not produced into an anterior angle, usually rounded anteriorly (for fig. see e.g. Ôhara 1994, fig. 3C)	**16**
16(21)	Protarsal groove deep, S-shaped (Fig. 46); body either cylindrical or depressed	**tribe Platysomatini Bickhardt, 1914**
17(18)	Body cylindrical (Fig. 8)	**Platysoma (Cylister) cornix Marseul, 1861**
18(17)	Body flattened (Fig. 6)	**19**
19(20)	Anterior angles of pronotum with dense punctures; pronotum on anterior third only slightly narrowed (Fig. 6)	**Platylister (Popinus) algiricus (Lucas, 1864)**
20(19)	Anterior angles of pronotum with sparse punctures; pronotum on anterior third narrowed more strongly (Fig. 7)	**Platylister (Popinus) simeani (Mulsant & Godart, 1875)** ^1^
21(16)	Prosternal groove usually shallow, not S-shaped (Fig. 47); body never cylindrical and usually only slightly flattened	**tribe Histerini Gyllenhal, 1808**
22(25)	Mesoventrite anteriorly outwardly arcuate, rounded (for fig. see e.g. Ôhara 1992, fig. 11D)	**23**
23(24)	Apical pronotal angles with a single stria; roundly-oval species (Figs 9, 121)	***Atholus duodecimstriatus duodecimstriatus* (Schrank, 1781)**
24(23)	Apical pronotal angles with double stria; a depressed taxon (Fig. 10)	***Eudiplister castaneus* (Ménétriés, 1832)**
25(22)	Mesoventrite deeply emarginate anteriorly (for fig. see e.g. Ôhara 1994, fig. 68C)	**26**
26(29)	Inner subhumeral stria completely absent (Fig. 12)	***Hister* Linnaeus, 1758**
27(28)	Elytra with red macula (Fig. 11)	***Hister limbatus* Truqui, 1852**
28(27)	Elytra completely black (Figs 12, 121)	***Hister sepulchralis* Erichson, 1834**
29(26)	Inner subhumeral stria present at least as a short fragment, usually complete (Fig. 14)	***Margarinotus* Marseul, 1854**
30(31)	Body large, PEL>7.50 mm, sub-rectangular; elytra usually with only striae I–III complete (Fig. 15)	**Margarinotus (Stenister) graecus graecus (Brullé, 1834)**
31(30)	Body smaller, PEL < 7.50 mm, roundly-oval; elytra with striae I–IV developed	**32**
32(33)	Pronotum with two lateral striae; a larger species, PEL > 4.50–7.00 mm (Figs 14, 121)	**Margarinotus (Ptomister) brunneus (Fabricius, 1775)**
33(32)	Pronotum with a single lateral stria; a smaller species, PEL = 2.80–4.00 mm (Figs 13, 121)	**Margarinotus (Grammostethus) ruficornis (Grimm, 1852)**
34(9)	Prosternum without prosternal lobe or “presternum” (for fig. see e.g. Ôhara 1994, fig. 12A)	**35**
35(36)	Tiny (PEL < 2.20 mm), completely black, dorsoventrally flattened subcortical taxon; elytra without striae (Fig. 18)	***Stenopleurum rothi* (Rosenhauer, 1856)**
36(35)	Usually larger (PEL > 2.20 mm), mostly metallic, occasionally with red macula, roundly-oval, not depressed taxa, never subcortical; elytra always striate (Fig. 19)	**subfamily Saprininae C.É. Blanchard, 1845**
37(38)	Frontal and supraorbital striae completely absent, basally between elytral stria IV and sutural elytral stria a short hooked appendix present (Fig. 22)	***Gnathoncus disjunctus suturifer* Reitter, 1896**
38(37)	At least supraorbital stria always present, frontal stria often interrupted medially, occasionally prolonged onto clypeus; without basal short hooked appendix between elytral stria IV and sutural stria	**39**
39(54)	Prosternal foveae present (Fig. 51)	**40**
40(41)	Carinal prosternal striae divergent anteriorly, “open”, lateral prosternal striae straight, terminating in deep prosternal foveae (Figs 23, 51, 122)	***Hemisaprinus subvirescens* (Ménétriés, 1832)**
41(40)	Carinal prosternal striae usually convergent and united anteriorly; lateral prosternal striae usually convergent anteriorly, occasionally surpassing prosternal foveae, in most cases evading them (for fig. see Lackner 2012, fig. 30)	**42**
42(43)	Underside of body setose, including elytral epipleuron; a very convex taxon; protibia with three large teeth topped by denticle, followed by five short denticles (Figs 43, 125)	***Xenonychus tridens* (Jacquelin du Val, 1853)**
43(42)	Underside of body usually glabrous, rarely pronotal hypomeron with very short setae (Hypocacculus (H.) metallescens)); elytral epipleuron always glabrous; slightly more flattened taxa; protibia usually with 3–8 short teeth topped by denticle, diminishing in size in proximal direction	**44**
44(49)	Frontal stria usually interrupted medially, slightly prolonged onto clypeus; if complete (*C. aemulus*) then elytral stria IV basally not united with sutural elytral stria	***Chalcionellus* Reichardt, 1932**
45(46)	Pronotum with pronotal post-ocular depressions; cuticle metallic, with bronze or slightly greenish hue (Fig. 20)	***Chalcionellus blanchii blanchii* (Marseul, 1855)**
46(45)	Pronotum without post-ocular pronotal depressions; cuticle not metallic, usually dark-brown or black (Fig. 21)	**47**
47(48)	Frontal stria weakened, but usually complete; elytral stria IV basally not connected with sutural elytral stria (Fig. 19)	***Chalcionellus aemulus* (Illiger, 1807)**
48 (47)	Frontal stria widely interrupted medially and prolonged onto clypeus; elytral stria IV basally connected with sutural elytral stria (Fig. 21)	***Chalcionellus libanicola* (Marseul, 1870)**
49(44)	Frontal stria usually complete; elytral stria IV usually basally united with sutural elytral stria	**genera *Hypocacculus* Bickhardt, 1914 and *Hypocaccus* C.G. Thomson, 1857**
50(53)	Frons with sparse minute punctures (Fig. 48)	***Hypocacculus* Bickhardt, 1914**
51(52)	Frontal stria medially almost straight, forming an acute angle above eyes; supraorbital stria keel-like (Figs 24, 48)	**Hypocacculus (Colpellus) praecox (Erichson, 1834)**
52(51)	Frontal stria medially outwardly arcuate, not forming an acute angle above eyes; supraorbital stria not keel-like (Figs 25, 49, 122)	**Hypocacculus (s.str.) metallescens (Erichson, 1834)**
53(50)	Frons densely and coarsely punctate, occasionally punctures forming coarse elongate rugae (Figs 26, 50)	**Hypocaccus (Nessus) baudii (Schmidt, 1890)**
54(39)	Prosternal foveae absent (Fig. 52)	***Saprinus* Erichson, 1834**
55(60)	Elytra bicolored (Fig. 34)	**56**
56(57)	At least the entire lateral elytral margin orange-red, usually most part of the elytral disk orange-red with only the short band along the elytral suture black (Fig. 34)	**Saprinus (S.) maculatus (P. Rossi, 1790)**
57(56)	Each elytron with a well-defined orange-red macula, never occupying the entire lateral elytral margin (Fig. 35)	**58**
58(59)	Black without bronze hue; macula reaching into fourth elytral interval (Fig. 35)	**Saprinus (S.) magnoguttatus Reichardt, 1926**
59(58)	Black with bronze hue; macula on elytron reaching into third elytral interval (Figs 31, 123)	**Saprinus (S.) externus (Fischer von Waldheim, 1823)**
60(55)	Elytra unicolored, never with red macula (Fig. 36)	**61**
61(62)	Pronotal hypomeron setose, fourth dorsal elytral stria strongly reduced, often absent; a large, usually metallic species (PEL = 5.00–7.50 mm) (Fig. 28)	**Saprinus (S.) caerulescens caerulescens (Hoffman, 1803)**
62(61)	Pronotal hypomeron asetose, fourth dorsal elytral stria usually not reduced, fully developed; smaller species (PEL = 2.50–6.50 mm)	**63**
63(64)	Elytra, especially their apical halves with very dense punctation, punctures aciculate and striolate, elytral intervals punctured, third dorsal elytral stria well-developed (Figs 39, 124)	***Saprinus* (*S* .) *strigil* Marseul, 1855**
64(63)	Elytra with variously dense punctation, but punctures usually not aciculate or striolate (some specimens of S. (S.) robustus can have striolate punctures, but then the third dorsal elytral stria is always strongly reduced) (Fig. 38)	**65**
65(70)	Elytra with well-defined polished areas ‘mirrors’, punctation of elytral disk very dense, punctures separated by less than their own diameter, third dorsal elytral stria reduced to absent (Fig. 32)	**66**
66(67)	Dorsal elytral striae erased by very coarse and dense punctures; pronotum with a well-defined ‘mirror’ consisting of three interconnected ovals of which the middle one is conspicuously larger than other two (Figs 32, 123)	***Saprinus* (S .) *figuratus* Marseul, 1855**
67(66)	Dorsal elytral striae always visible; pronotum without a well-defined “mirror” (Fig. 27)	**68**
68(69)	Elytral ‘mirror’ with microscopic scattered punctation, light to dark brown species, without greenish or bronze metallic hue, third dorsal elytral stria reduced, but usually discernible; elytral punctation in fourth elytral interval reaches elytral half (Fig. 27)	***Saprinus* (S .) *aegialius* Reitter, 1884**
69(68)	Elytral ‘mirror’ glabrous, third dorsal elytral stria usually strongly reduced to absent, dorsum with distinct greenish or bronze metallic hue; punctation in fourth elytral interval does not reach elytral half (Fig. 37)	**Saprinus (S.) prasinus prasinus Erichson, 1834**
70(65)	Elytra without well-defined polished areas (‘mirrors’), punctation of the elytral disk less dense, punctures usually separated by their own diameter or more (Fig. 36)	**71**
71(72)	Apices of carinal prosternal striae convergent anteriorly, rather approximate; large (PEL = 4.50–6.50 mm) entirely black species (Figs 36, 52, 123)	**Saprinus (S.) niger Motschulsky, 1849**
72(71)	Apices of carinal prosternal striae divergent anteriorly (Fig. 53)	**73**
73(76)	Apices of carinal prosternal striae strongly divergent, laying on lateral sides of the prosternal process (Fig. 53); usually moderately large, brownish species (PEL = 3.50–5.30 mm)	**74**
74(75)	Pronotal post-ocular depressions deep, third dorsal elytral stria usually not reduced, light to dark brown species with slight bronze metallic hue (Fig. 41), male with deeply depressed metaventrite; male terminalia: apex of 8^th^ sternite (velum) asetose, 8^th^ sternite medially not strongly sclerotized (Figs 57–65, 125)	**Saprinus (S.) subnitescens Bickhardt, 1909**
75(74)	Pronotal post-ocular depressions shallow, third dorsal elytral stria usually strongly reduced, black species without metallic hue (Fig. 38), male with only shallowly depressed metaventrite; male terminalia: apex of 8^th^ sternite (velum) with dense tiny setae, 8^th^ sternite medially strongly sclerotized (Figs 66–74, 124)	**Saprinus (S.) robustus Krása, 1944**
76(73)	Apices of prosternal striae divergent, but never laying on lateral sides of the pronotal process (Fig. 54); usually smaller species (PEL= 2.50–3.90 mm)	**77**
77(78)	Pronotal post-ocular depressions absent, pronotal disk medially with distinct punctation, humeral elytral stria confluent with inner subhumeral one creating a supplementary dorsal elytral stria parallel to first (Fig. 33); male terminalia: apices of 8^th^ sternite with thin, dense brush of setae, medio-laterally with a bean-shaped setose sclerite, aedeagus strongly constricted before apex (Figs 75–83, 123)	**Saprinus (S.) godet (Brullé, 1832)**
78(77)	Pronotal post-ocular depressions present, pronotal disk medially with only scattered fine punctation (Fig. 42)	**79**
79(80)	Entire elytral disk with punctation, punctures separated by twice or more their diameter, dorsal elytral striae thin, impunctate (Fig. 42), antennal club large, light-amber coloured; male terminalia: apices of 8^th^ sternite with tiny triangular accessory sclerite furnished with micro-setae, aedeagus short and stout, not dilated apically (Figs 84–92, 125)	***Saprinus* (*S* .) *tenuistrius sparsutus* Solsky, 1876**
80(79)	At least the area between united sutural and fourth dorsal elytral striae without punctation (or punctures microscopic), punctures of elytral disk separated usually by less than twice their diameter (Fig. 40), antennal club medium-sized, reddish-brown. The following species are usually only reliably identifiable based on their male terminalia	**81**
81(82)	Apical margin of metaventrite of male without tubercles. Male terminalia: 8^th^ sternite with two rows of brush-like setae: one situated approximately medially and another apically, aedeagus constricted before apex; apex rounded (Figs 93–101) (Figs 40, 54)	**Saprinus (S.) submarginatus J. Sahlberg, 1913**
82(81)	Apical margin of metaventrite of male with two distinct tubercles (Fig. 55)	**83**
83(84)	Tubercles on the apical margin of metaventrite of male slightly removed from metaventral margin (Fig. 55). Dorsal elytral striae surpassing elytral half; male terminalia: 8^th^ sternite with large setose velum (best seen especially from lateral view), apex of aedeagus rectangularly dilated, truncated (Figs 102–110) (Figs 29, 122)	***Saprinus* (*S* .) *calatravensis* Fuente, 1899**
84(83)	Tubercles situated almost on the very apical metaventral margin (Fig. 56); dorsal elytral striae usually not surpassing elytral half; male terminalia: 8^th^ sternite without large setose velum, apex of aedeagus only slightly roundly dilated (Figs 111–119) (Figs 30, 122)	**Saprinus (S.) chalcites (Illiger, 1807)**

## Checklist of the Histeridae of Lebanon and surrounding countries

This checklist is based on Lackner et al. (2015) as the main reference; other relevant sources of information included Mazur (2011) and Shayya et al. (2018) (Table 2).

**Table 2.** Checklist of the Histeridae of Lebanon and surrounding countries.

Species	Lebanon	Syria	Israel	Cyprus
*Abraeomorphus besucheti* Mazur, 1977	X		X	
*Abraeomorphus minutissimus* (Reitter, 1884)	X			
Acritus (Acritus) nigricornis (Hoffmann, 1803)			X	
Acritus (Acritus) minutus (Herbst, 1791)				X
Acritus (Pycnacritus) homoeopathicus Wollaston, 1857				X
*Alienocacculus vanharteni* Kanaar, 2008			X	
*Anapleus raddei* (Reitter, 1877)			X	
*Anapleus wewalkai* Olexa, 1982		X		X
*Atholus bimaculatus* (Linnaeus, 1758)		X	X	X
*Atholus corvinus* (Germar, 1817)		X	X	
*Atholus duodecimstriatus duodecimstriatus* (Schrank, 1781)	X	X	X	
*Atholus scutellaris* (Erichson, 1834)		X	X	X
*Carcinops pumilio* (Erichson, 1834)				X
Chaetabraeus (Chaetabraeus) lucidus (Peyerimhoff, 1917)		X		
Chaetabraeus (Mazureus) convexus (Reitter, 1884)		X	X	
*Chalcionellus aemulus* (Illiger, 1807)	X		X	
*Chalcionellus amoenus* (Erichson, 1834)		X		
*Chalcionellus blanchii blanchii* (Marseul, 1855)	X	X	X	X
*Chalcionellus decemstriatus decemstriatus* (P. Rossi, 1792)		X	X	X
*Chalcionellus libanicola* (Marseul, 1870)	X	X		
*Chalcionellus mersinae* (Marseul, 1857)		X		
*Chalcionellus palaestinensis* (Schmidt, 1890)		X	X	
*Chalcionellus tunisius* (Marseul, 1875)		X		
*Chalcionellus turcicus* (Marseul, 1857)		X		
*Chalcionellus tyrius* (Marseul, 1857)		X		X
*Epierus comptus* (Erichson, 1834)		X		
*Eudiplister castaneus* (Ménétriés, 1832)	X	X	X	X
*Eudiplister peyroni* (Marseul, 1857)		X	X	
*Eudiplister planulus* (Ménétriés, 1849)		X	X	
*Gnathoncus disjunctus suturifer* Reitter, 1896	X	X		
*Gnathoncus rotundatus* (Kugelann, 1792)		X	X	
*Hemisaprinus cyprius* Dahlgren, 1981				X
*Hemisaprinus subvirescens* (Ménétriés, 1832)	X*	X	X	X
*Hister bipunctatus* Paykull, 1811				X
*Hister hanka* Kapler, 1994				X
*Hister illigeri reductus* G. Müller, 1960		X	X	
*Hister judaicus* Mazur, 2008			X	
*Hister limbatus* Truqui, 1852	X	X		
*Hister lugubris* Truqui, 1852				X
*Hister quadrimaculatus* Linnaeus, 1758				X
*Hister sepulchralis* Erichson, 1834	X*	X		
Hypocacculus (Colpellus) biskrensis (Marseul, 1876)		X		
Hypocacculus (Colpellus) praecox (Erichson, 1834)	X	X	X	X
Hypocacculus (Hypocacculus) atrocyaneus (J. Schmidt, 1888)		X		
Hypocacculus (Hypocacculus) metallescens (Erichson, 1834)	X	X	X	X
Hypocaccus (Hypocaccus) brasiliensis (Paykull, 1811)			X	X
Hypocaccus (Hypocaccus) crassipes (Erichson, 1834)		X		
Hypocaccus (Nessus) baudii (J. Schmidt, 1890)	X	X	X	X
Hypocaccus (Nessus) interpunctatus interpunctatus (J. Schmidt, 1885)		X		
Hypocaccus (Nessus) japhonis (J. Schmidt, 1890)		X	X	
Hypocaccus (Nessus) rubripes (Erichson, 1834)		X		X
Hypocaccus (Nessus) curtus (Rosenhauer, 1847)				X
Margarinotus (Eucalohister) kurdistanus kurdistanus (Marseul, 1857)		X	X	
Margarinotus (Grammostethus) ruficornis (Grimm, 1852)	X	X	X	
Margarinotus (Paralister) carbonarius carbonarius (Hoffmann, 1803)		X	X	X
Margarinotus (Paralister) carbonarius macedonicus (J. Müller, 1937)			X	
Margarinotus (Paralister) purpurascens (Herbst, 1791)		X		
Margarinotus (Ptomister) brunneus (Fabricius, 1775)	X		X	
Margarinotus (Ptomister) integer (Brisout de Barneville, 1866)			X	
Margarinotus (Stenister) graecus graecus (Brullé, 1832)	X	X	X	X
Margarinotus (Stenister) graecus horni (Bickhardt, 1912)		X	X	
Margarinotus (Stenister) obscurus (Kugelann, 1792)		X	X	
*Merohister ariasi* (Marseul, 1864)		X	X	
*Notodoma lewisi* Reitter, 1910	X	X		
*Onthophilus affinis* L. Redtenbacher, 1847		X	X	
*Onthophilus bickhardti* Reitter, 1909	X		X	
*Onthophilus convictor* Normand, 1919		X		
*Onthophilus punctatus caucasicus* Reitter, 1890			X	
*Onthophilus punctatus punctatus* (O.F. Müller, 1776)		X		
*Onthophilus striatus inconditus* Reichardt, 1941	X	X	X	X
Pachylister (Pachylister) inaequalis (Olivier, 1789)		X		X
*Pactolinus major* (Linnaeus, 1767)		X	X	X
*Paravolvulus syphax* (Reitter, 1904)		X		
*Pholioxenus kodymi* Olexa, 1984		X		
*Pholioxenus krali* Olexa, 1984		X		
Platylister (Popinus) simeani (Mulsant & Godart, 1875)	X			
*Platylomalus complanatus* (Panzer, 1797)		X		X
Platysoma (Cylister) cornix Marseul, 1861	X	X	X	X
Platysoma (Platysoma) compressum (Herbst, 1783)		X		
Platysoma (Platysoma) inexpectatum Lackner, 2004		X		
Plegaderus (Plegaderus) otti Marseul, 1856			X	
Plegaderus (Hemitrichoderus) adonis Marseul, 1876		X		X
Saprinus (Phaonius) pharao Marseul, 1855		X	X	X
Saprinus (Saprinus) acuminatus acuminatus (Fabricius, 1798)		X		
Saprinus (Saprinus) aegialius Reitter, 1884	X	X		
Saprinus (Saprinus) aeneus (Fabricius, 1775)		X		
Saprinus (Saprinus) caerulescens caerulescens (Hoffmann, 1803)	X	X	X	X
Saprinus (Saprinus) calatravensis Fuente, 1899	X	X	X	
Saprinus (Saprinus) chalcites (Illiger, 1807)	X		X	X
Saprinus (Saprinus) algericus (Paykull, 1811)				X
Saprinus (Saprinus) concinnus (Gebler, 1830)		X		
Saprinus (Saprinus) delta Marseul, 1862		X		
Saprinus (Saprinus) externus (Fischer von Waldheim, 1823)	X*	X		
Saprinus (Saprinus) figuratus Marseul, 1855	X*	X	X	
Saprinus (Saprinus) georgicus Marseul, 1862		X	X	
Saprinus (Saprinus) godet (Brullé, 1832)	X	X	X	
Saprinus (Saprinus) intractabilis Reichardt, 1929		X		
Saprinus (Saprinus) maculatus (P. Rossi, 1792)	X	X	X	X
Saprinus (Saprinus) magnoguttatus Reichardt, 1926	X	X		
Saprinus (Saprinus) moyses Marseul, 1862		X		
Saprinus (Saprinus) niger Motschulsky, 1849	X*	X	X	
Saprinus (Saprinus) ornatus Erichson, 1834		X	X	
Saprinus (Saprinus) planiusculus Motschulsky, 1849		X		
Saprinus (Saprinus) politus politus (Brahm, 1790)		X	X	
Saprinus (Saprinus) prasinus aeneomicans G. Müller, 1960		X	X	
Saprinus (Saprinus) prasinus prasinus Erichson, 1834	X	X		X
Saprinus (Saprinus) robustus Krása, 1944	X	X	X	X
Saprinus (Saprinus) ruber gemmingeri Marseul, 1864		X	X	
Saprinus (Saprinus) semistriatus (Scriba, 1790)			X	
Saprinus (Saprinus) sinaiticus Crotch, 1872		X	X	
Saprinus (Saprinus) strigil Marseul, 1855	X	X	X	X
Saprinus (Saprinus) stussineri Reitter, 1909			X	
Saprinus (Saprinus) submarginatus J. Sahlberg, 1913	X	X	X	
Saprinus (Saprinus) subnitescens Bickhardt, 1909	X	X	X	X
Saprinus (Saprinus) tenuistrius sparsutus Solsky, 1876	X	X	X	X
*Spathochus coyei* Marseul, 1864		X	X	X
*Stenopleurum rothi* (Rosenhauer, 1856)	X	X		X
*Sternocoelis diversepunctatus* Pic, 1911				X
*Sternocoelis robustus* Pic, 1911				X
Teretrius (Teretrius) accaciae Reitter, 1900			X	
Teretrius (Teretrius) fabricii Mazur, 1972		X	X	
Teretrius (Teretrius) pulex Fairmaire, 1877		X		
Tribalus (Tribalus) sp.	X*	X	X	
Tribalus (Tribalus) anatolicus Olexa, 1980				X
Tribalus (Tribalus) scaphidiformis (Illiger, 1807)				X
*Xenonychus tridens* (Jacquelin du Val, 1853)	X*	X	X	X
*Zorius exilis* Reichardt, 1932			X	
*Zorius funereus* (Schmidt, 1890)			X	

* – newly reported from Lebanon

## Discussion

In general, faunistic inventories are incomplete since the number of studied species continues to increase with the increase of sampling efforts (Baz et al. 2014). Thus, this paper enriches the knowledge of the fauna of Histeridae in Lebanon as 41 species are currently known from Lebanon. In addition to the knowledge of *Saprinus* of forensic relevance (Shayya et al. 2018), our study lists species and their biology for 17 other genera: *Abraeomorphus*, *Atholus*, *Chalcionellus*, *Eudiplister*, *Gnathoncus*, *Hemisaprinus*, *Hister*, *Hypocaccus*, *Hypocacculus*, *Margarinotus*, *Notodoma*, *Onthophilus*, *Platylister*, *Platysoma*, *Stenopleurum*, *Tribalus*, and *Xenonychus*. It furthermore reports different sampling efforts and mentions six new records for the fauna of the country. The key provided in this study provides a foundation for the identification of histerids from Lebanon for other entomological and ecological studies in the country. The following are comments on species biology and some implications.

### Species attracted to ephemeral microhabitats that could be of forensic relevance

*Saprinus* and *Margarinotus* are dominant genera on ephemeral and unstable microhabitats like carcasses, dung, and decaying plants (Mazur 1981; Bajerlein et al. 2011). Many species belonging to these two genera are considered eurytopic, able to tolerate a wide range of microhabitats (Bajerlein 2011). Species among these genera were found to oviposit their eggs near the carcass, where there is no larval mass and the soil temperature is cooler (Bajerlein et al. 2011; Caneparo et al. 2017). Saprinus (S.) subnitescens was the most abundant species on pig carrion (Shayya et al. 2018). In our samplings, this species was also abundant on rotting fish-baited pitfall traps especially in Shouf-Aley region and it was also present on dung in Kfeir. Also, Saprinus (S.) strigil was common on decomposing pig carcasses (Shayya et al. 2018). It was also associated with other ephemeral microhabitats used in this study. Saprinus (S.) chalcites, Saprinus (S.) godet, Saprinus (S.) robustus, Saprinus (S.) tenuistrius sparsutus were also attracted to dung, decomposing fish and to carrion (Shayya et al. 2018).

Saprinus (S.) caerulescens was only attracted to mammalian carrion and it was absent from other baits (rotting fish and dung). This is in accordance with T. Lackner’s personal observation that this species is common on large carcasses. However, Kryzhanovskij and Reichardt (1976) collected this species on rotten fish, where it preyed upon dermestids, whereas Anlaş et al. (2007) collected it from cow dung in Turkey. The preference of this species to a specific microhabitat should be further investigated in future studies.

*Margarinotus* spp. are varied in habits (Caterino 2010). In our samplings (Mediterranean climate), they were present on carrion only in spring; absent in summer. They were less frequent in rotting fish-baited pitfall traps. Despite the differences in climate, this is in accordance with Bajerlein et al. (2011), who found that their abundance was highest in spring, and decreased markedly in summer in a study in West Poland, a temperate climate. Also, Kočárek’s (2003) work supported the highest abundance of histerids in spring during a study in the warm and temperate climate of Opava, Czech Republic. *Margarinotus* likewise shows preference to cold and humid forest floor (Bajerlein et al. 2011). The latter condition is common especially in early spring in Lebanon (Abi Saab et al. 2019).

*Atholus duodecimstriatus duodecimstriatus* was collected from carrion only during summer. Unlike the findings of Bajerlein (2009) and the review of Kovarik and Caterino (2016), which mentioned the preference of this species to dung, it was absent from other ephemeral microhabitats of this study. Similarly, Hypocacculus (H.) metallescens was rare and collected during summer from carrion and absent from other decomposing matter, dung, and sand dunes, which it is normally associated with (Penati 2009). Thus, more sampling efforts should be made to elucidate the habitat preference of these two species. *Hister* is mainly found in cattle and horse dung (Rozner 2010). This is in accordance with our study as *Hister sepulchralis* was absent from carrion and only attracted to pig dung. However, the arrival of *Hister* spp. on carrion during the active decay stage and their presence in the advanced decay and dry stages, as reported by Wolff et al. (2001), was not observed during our sampling from decomposing carrion in Lebanon. *Chalcionellus* spp. were previously recorded from Lebanon, but absent from our collecting. They are usually attracted to excrements and decomposing carcasses (Kryzhanovskij and Reichardt 1976).

According to Nuorteva (1970), histerids, especially those among the genera *Saprinus* and *Hister* are attracted to fish carcasses later than blowflies, predate on fly egg and larvae and even destroy the full-grown fly larvae. Thus, there is a negative correlation between the occurrence of blowflies and histerids on fish carcasses (Nuorteva 1970). In our collections, Saprinus (S.) figuratus was found only on fish carcasses and not on pig ones. In a similar Mediterranean climate, in Spain, this species was found to be rare and restricted to mesomediterranean holm oak forests (Martín-Vega et al. 2015). Unlike the findings of Reichardt (1941), Saprinus (S.) niger was also collected only from fish carcasses and not from other carrion. In Spain, it was found also on squid carcasses, which are effective for collecting carrion insects and species inventories resemble those from pig studies (Martín-Vega et al. 2015). Al-Tunsoy et al. (2017) suggested that Saprinus (S.) externus is uncommon in Turkey and that carrion is not its primary habitat. Also, Rozner (2010) mentioned that it is very rare. In our samplings we found two specimens on dung and fish but none on pig carrion.

### Species collected from humid habitats

The subgenus Tribalus s. str. of the genus *Tribalus* is a species-rich group mostly occurring in Africa with smaller number of species found in the Palaearctic and Oriental regions (Mazur 2011; Lackner and Vienna 2017). Similar to the findings Lackner and Vienna (2017), we found this genus under stones in wetter areas on the riverside of Baissour and Rechmaya rivers and also in the soil detritus at Rechmaya riverside. Also, Lackner and Chehlarov (2006) found it in pitfall traps on the right shore of Struma River in Bulgaria. Kovarik and Caterino (2016) mentioned that this genus is attracted to organic material beneath old dead trees and to rotting wood. They likewise mentioned that Tribalus (T.) capensis (Paykull, 1811) shows preference to dung that has lost much of its moisture and that Tribalus (T.) cavernicola Lewis, 1908 occurs in cave entrances. Those two species of *Tribalus* are not found in the Palaearctic region (Lackner et al. 2015). The subgenus contains many undescribed, externally rather uniform species that can be most-reliably separated based on their male genitalia (Lackner and Vienna 2017). It is therefore and due to the lack of reliable taxonomic keys we didn’t assign the exact taxonomic identification and we advocate its revision.

### Ultra-psammophilic species

The genus *Xenonychus* is an ultra-psammophilic group, which is found exclusively in sand dune systems. They are buried in sand and known as sand-swimmers. They can be found by laborious methods near the roots of plants (Lackner et al. 2019). According to the personal observation of T.L., members of *Xenonychus* could be found on carrion that is on sand or buried in it. Also, Hypocacculus (C.) praecox is a psammo-halobiotic species (Penati 2009). We found *Xenonychus tridens* on a sandy beach in Tyre (Lebanon). It is important to study the entomofauna associated with carcasses on the seashore or those buried in sand, which could help in estimating the minimum postmortem interval (PMI_min_). For instance, in 2018 a murdered woman was found covered with sand in Sidon Rmeileh beach-Lebanon (Zaatari 2018).

### Species attracted to decaying plants, trees bark, and fungi

*Abraeomorphus minutissimus*, *Eudiplister castaneus*, Margarinotus (Grammostethus) ruficornis, Platysoma (Cylister) cornix, and Platylister (Popinus) simeani are often associated with decaying plant matter and/or bark of trees like pine (Kryzhanovskij and Reichardt 1976; Kovarik and Caterino 2016). *Notodoma lewisi* is a fungivorous species. Fungi can provide insects with nutrients and essential elements, and in recently dead wood they detoxify plant defenses and provide protection (Birkemoe et al. 2018). Such beetles might serve as a dispersal vector for dead-wood-inhabiting fungi. More knowledge on such interaction is detailed in Seibold et al. (2019).

## Conclusions

This is considered the first faunistic study of the Histeridae family in Lebanon with a key to all locally recorded species, comments on their biology, and possible implications in the country of study. More ecological research should be performed in different Lebanese regions and more quantitative data are needed to clarify the habitat preference of Histeridae species. Different sampling methods such as sifting, Flight Interception Traps (FIT), pitfall traps, etc. should be also used in the future. Seasonal sampling and replicates are needed to show the peak activity, seasonality, and habitat preferences of members of this family.

## Footnotes

According to Y. Gomy (Nevers, France; personal communication, 2019) the two species of Platylister are possibly synonyms. This has already been discussed by Kanaar (2008); according to him “A comparison of the type-specimens of both species is needed to solve this question”.

## References

[B1] Abi Saab MT Sellami MH Giorio P Basile A Bonfante A Rouphael Y Fahed S Jomaa I Stephan C Kabalan R Massaad R Todorovic M Albrizio R (2019) Assessing the potential of cereal production systems to adapt to contrasting weather conditions in the Mediterranean Region. Agronomy 9(393): 21 pp. 10.3390/agronomy9070393

[B2] Al-Tunsoy F Turan Y Firat S Sert O (2017) Differences in succession of Coleoptera species attracted to pig carcasses in rural and urban habitats in Eskişehir Province, Turkey. Türkiye Entomoloji Dergisi 41(2): 177–195. 10.16970/ted.65825

[B3] Amendt J Richards CS Campobasso CP Zehner R Hall MJR (2011) Forensic entomology: applications and limitations. Forensic Science Medicine and Pathology 7(4): 379–392. 10.1007/s12024-010-9209-2 21213072

[B4] Anlaş S Lackner T Tezcan S (2007) A cow dung investigation on Histeridae (Coleoptera) with a new record for Turkey. Baltic Journal of Coleopterology 7(2): 157–164.

[B5] Bajerlein D (2009) Coprophilous histerid beetle community (Coleoptera: Histeridae) of western Poland. Polish Journal of Entomology 78: 201–207.

[B6] Bajerlein D Matuszewski S Konwerski S (2011) Insect succession on carrion: seasonality, habitat preference and residency of histerid beetles (Coleoptera: Histeridae) visiting pig carrion exposed in various forests (Western Poland). Polish Journal of Ecology 59(4): 787–797.

[B7] Barbosa TM Vasconcelos SD (2018) Muscidae (Diptera) of medico-legal importance associated with ephemeral organic substrates in seasonally dry tropical forests. Papeis. Avulsos de Zoologia 58: 1–4 e20185826. 10.11606/1807-0205/2018.58.26

[B8] Baz A Cifrián B Martín-Vega D (2014) Patterns of diversity and abundance of carrion insect assemblages in the natural park “Hoces del Río Riaza” (Central Spain). Journal of Insect Science 14(162): 10 pp. 10.1093/jisesa/ieu024 PMC5443602 25368080

[B9] Birkemoe T Jacobsen RM Sverdrup-Thygeson A Biederman PHW (2018) Insect-fungus interactions in dead wood systems. In: Ulyshen MD (Ed.) Saproxylic Insects, Zoological Monographs 1. 10.1007/978-3-319-75937-1_12

[B10] Borghesio L Penati F Palestrini C (2002) Hister beetles of a site in the pre-Apennines of Piedmont (Italy) (ColeopteraHisteridae). Bollettino della Società entomologica italiana 134(2): 99–110.

[B11] Bousquet Y Laplante S (2006) ColeopteraHisteridae. The Insects and Arachnids of Canada Part 24. NRC Research Press, Ottawa, xiii + 485 pp

[B12] Cajaiba RL Périco E Silva WB Santos M (2017) Seasonal patterns in the diversity of histerid beetles (Histeridae) are ecosystem specific? A case in Para state, northern Brazil. Applied Ecology and Environmental Research 15: 1227–1237. 10.15666/aeer/1504_12271237

[B13] Caneparo MFC Fischer ML Almeida LM (2017) Effect of temperature on the life cycle of *Euspilotus azureus* (Coleoptera: Histeridae), a predator of forensic importance. Florida Entomologist 100(4): 795–801. 10.1653/024.100.0404

[B14] Caterino M (2010) A review of California *Margarinotus* Marseul (Coleoptera: Histeridae: Histerinae: Histerini), with descriptions of two new species. The Coleopterists’ Bulletin 64(1): 1–12. 10.1649/0010-065X-64.1.1

[B15] Caterino MS Tishechkin AK (2014) New genera and species of Neotropical Exosternini (Coleoptera: Histeridae). ZooKeys 381: 11–78. https://zookeys.pensoft.net/article/3369/10.3897/zookeys.381.6772 PMC3950425 24624014

[B16] Carlton CE Leschen RAB Kovarik PW (1996) Predation on adult blow flies by a Chilean hister beetle, *Euspilotus bisignatus* (Erichson) (Coleoptera: Histeridae). The Coleopterists’ Bulletin 50(2): 154.

[B17] Davis ALV (1994) Associations of Afrotropical Coleoptera (Scarabaeidae, Aphodiidae, Staphylinidae, Hydrophilidae, Histeridae) with dung and decaying matter: implications for selection of fly-control agents for Australia. Journal of Natural History 28: 383–399. 10.1080/00222939400770171

[B18] Fagundes CK Di Mare RA Wink C Manfio D (2011) Diversity of the families of Coleoptera captured with pitfall traps in five different environments in Santa Maria, RS, Brazil. Brazilian Journal of Biology 71: 381–390. 10.1590/S1519-69842011000300007 21755155

[B19] Gimmel ML Ferro ML (2018) Chapter 2: General Overview of saproxylic Coleoptera. In: Ulyshen MD (Ed.) Saproxylic Insects.Zoological Monographs, Springer Nature, city, 51–128. 10.1007/978-3-319-75937-1_2

[B20] Goff ML (1993) Estimation of postmortem interval using arthropod development and successional patterns. Forensic Science Review 5(2): 81–94. 26270076

[B21] Goff M (2009) Early postmortem changes and stages of decomposition in exposed cadavers. Experimental and applied acarology 49: 21–36. 10.1007/s10493-009-9284-9 19554461

[B22] Kanaar P (1997) Revision of the genus *Paratropus* Gerstaecker (Coleoptera: Histeridae). Zoologische Verhandelingen 315: 1–183.

[B23] Kanaar P (2008) Order Coleoptera, family Histeridae. In: Harten A van (Ed.) Arthropod Fauna of the United Arab Emirates.Volume 1. Multiply Marketing Consultancy Services, Abu Dhabi, 170–193.

[B24] Khater C El-Hajj R (2012) Terrestrial biodiversity in Lebanon. National Council for Scientific Research, Lebanon, 141–169.

[B25] Kočárek P (2003) Decomposition and Coleoptera succession on exposed carrion of small mammal in Opava, the Czech Republic. European Journal of Soil Biology 39: 31–45. 10.1016/S1164-5563(02)00007-9

[B26] Kovarik PW Caterino MS (2016) Histeridae. In: Beutel RG Leschen RAB (Eds) Handbook of Zoology Part 38, Coleoptera, Vol.1: Morphology and Systematics (2nd edn). Walter de Gruyter, Berlin, 275–314.

[B27] Kryzhanovskij OL Reichardt AN (1976) Zhuki Nadsemeystva Histeroidea (semeystva Sphaeritidae, Histeridae, Synteliidae). [Beetles of the superfamily Histeroidea (families Sphaeritidae, Histeridae, Synteliidae)]. Fauna SSSR, Zhestokrylye, Vyp. 4. Nauka, Leningrad, 434 pp. [In Russian]

[B28] Lackner T (2009) First records of *Spathochus coeyi* from Cyprus and Teretrius (Neotepetrius) parasita from Greece (Coleoptera: Histeridae). Klapalekiana 45: 73–74.

[B29] Lackner T (2010) Review of the Palaearctic genera of Saprininae (Coleoptera: Histeridae). Acta Entomologica Musei Nationalis Pragae 50 (Supplementum): 1–254.

[B30] Lackner T (2012) Revision of the genus *Xenonychus* Wollaston, 1864. Acta Entomologica Musei Nationalis Pragae 52(1): 147–159.

[B31] Lackner T (2014a) Phylogeny of the Saprininae reveals interesting ecological shifts in the history of the subfamily (Coleoptera: Histeridae). Zoological Journal of the Linnean Society 172: 521–555. 10.1111/zoj12182

[B32] Lackner T (2014b) Revision of the genus *Hemisaprinus* Kryzhanovskij, 1976 (Coleoptera, Histeridae, Saprininae). ZooKeys 429: 101–130. 10.3897/zookeys.429.7949 PMC4137301 25147473

[B33] Lackner T (2015) Coleoptera: Sphaeritidae, Histeridae. Folia Heyrovskyana, Series B 23: 1–33.

[B34] Lackner T (2020) A review of Gnathoncus of Southeast Asia (Coleoptera: Histeridae: Saprininae). Acta Entomologica Musei Nationalis Pragae 60(1): 397–409. 10.1002/mmnd.48018960133

[B35] Lackner T Hlaváč P (2002) A new record of *Notodoma lewisi* from Turkey. Entomological Problems 32(2): 165–166.

[B36] Lackner T Mazur S Newton AF (2015) Family Histeridae. In: Löbl I Löbl D (Eds) Catalogue of Palaearctic Coleoptera.Vol. 2. Hydrophiloidea – Staphylinoidea, part 1. Brill Publishers, Leiden, Boston, 76–130.

[B37] Lackner T Leschen RAB (2017) A monograph of the Australopacific Saprininae (Coleoptera, Histeridae). ZooKeys 738: 1–263. 10.3897/zookeys.689.12021.figure263 PMC5672588 29200920

[B38] Lackner T Vienna P (2017) Histeridae of Socotra (Coleoptera: Histeroidea). Entomologica Musei Nationalis Pragae 57 (Supplementum): 55–76. 10.1515/aemnp-2017-0107

[B39] Lackner T Tarasov S (2019) Female genitalia are moderately informative for phylogenetic inference and not concerted with male genitalia in Saprininae beetles (Coleoptera: Histeridae). Systematic Entomology. 10.1111/syen.12346

[B40] Lackner T Kindler C Motyka M Balke M (2019) Molecular phylogeny of the Saprininae (Coleoptera:Histeridae): the evolution of psammophily or life in sand. Biological Journal of the Linnean Society 20: 1–13. 10.1093/biolinnean/blz011

[B41] Martín-Vega D Cifrián B Díaz-Aranda LM Baz A (2015) Necrophilous histerid beetle communities (Coleoptera: Histeridae) in Central Spain: species composition and habitat preferences. Environmental Entomology 44(4): 966–974. 10.1093/ee/nvv077 26314042

[B42] Matuszewski S Bajerlein D Konwerski S Szpila K (2008) An initial study of insect succession and carrion decomposition in various forest habitats of Central Europe. Forensic Science International 180: 61–69. 10.1016/j.forsciint.2008.06.015 18715728

[B43] Matuszewski S Szafałowicz M (2013) Temperature-dependent appearance of forensically useful beetles on carcasses. Forensic Science International 229: 92–99. 10.1016/j.forsciint.2013.03.034 23683913

[B44] Mazur S (1981) Histeridae-gnilikowate (Insecta: Coleoptera) Fauna of Poland. Polish Entomological Society, Warszawa, 206 pp. [In Polish]

[B45] Mazur S (2011) A Concise catalogue of the Histeridae (Insecta: Coleoptera). Warsaw University of Life Sciences, 332 pp.

[B46] Mazur S Górz A Byk A (2017) Coprophilous histerids (Coleoptera: Histeridae) of the Polish Carpathians. Fragmenta Faunistica 60(1): 15–22.

[B47] Nuorteva P (1970) Histerid beetles as predators of blowflies (Diptera, Calliphoridae) in Finland. Annales Zoologici Fennici 7: 195–198.

[B48] Ôhara M (1992) A revision of the Japanese species of the genus *Atholus* (Coleoptera, Histeridae), Part 1. Elytra (Tokyo) 20(2): 167–182.

[B49] Ôhara M (1994) A revision of the superfamily Histeroidea of Japan (Coleoptera). Insecta Matsumurana (NS) 51: 1–238.

[B50] Özdemir S Sert O (2008) Systematic studies on male genitalia of Coleoptera species found on decomposing pig (*Sus Scrofa* L.) carcasses at Ankara Province. Hacettepe Journal of Biology and Chemistry 36(2): 137–161.

[B51] Payne JA (1965) A summer carrion study of the baby pig *Sus Scrofa* Linnaeus. Ecology 46(5): 592–602. 10.2307/1934999

[B52] Penati F (2009) An updated catalogue of the Histeridae (Coleoptera) of Sardinia, with faunistic, zoogeographical, ecological and conservation remarks. Zootaxa 2318: 197–280. 10.11646/zootaxa.2318.1.8

[B53] Pinheiro J (2006) Decay Process of a Cadaver. In: Schmitt A Cunha E Pinheiro J (Eds) Forensic Anthropology and Medicine, Complementary Sciences from Recovery to Cause of Death.Humana Press, Totowa, 85–116. 10.1007/978-1-59745-099-7_5

[B54] Polat A Yıldırım E (2017) A contribution to the knowledge of the Histeridae (Coleoptera) fauna of Turkey. Linzer Biologische Beiträge 49(2): 1523–1527.

[B55] Reichardt A (1941) Semeystva Sphaeritidae i Histeridae (Vol. 1). [Families Sphaeritidae and Histeridae]. In: Fauna SSSR, Nasekomye Zhestokrylye, V, 3. Nauka, Moskva-Leningrad, xiii + 419 pp. [In Russian]

[B56] Reitter E (1910) Ein neuer palearctischer Vertreter der ostindisch-japanischen Histeridengattung *Notodoma* Mars. aus Hochsyrien. Entomologischen Blättern 6: 164–165. 10.5962/bhl.part.23347

[B57] Rozner I (2010) Additional data to the hister beetle fauna of Turkey (Coleoptera: Histeridae). Natura Somogyiensis 17: 171–176.

[B58] Sanchez A Chittaro Y (2018) Liste commentée des Histeridae et Sphaeritidae de Suisse (Coleoptera, Histeroidea). [Annotated list of Histeridae and Sphaeritidae from Switzerland (Coleoptera, Histeroidea)]. Entomologische Blätter und Coleoptera 114: 335–352. [In French]

[B59] Seibold S Müller J Baldrian P Cadotte MW Štursová M Biedermann PHW Krah FS Bässler C (2019) Fungi associated with beetles dispersing from dead wood-let’s take the beetle bus! Fungal Ecology 39: 100–108. 10.1016/j.funeco.2018.11.016

[B60] Shayya S Dégallier N Nel A Azar D Lackner T (2018) Contribution to the knowledge of *Saprinus* Erichson, 1834 of forensic relevance from Lebanon (Coleoptera, Histeridae). ZooKeys 738: 117–152. 10.3897/zookeys.738.21382 PMC5904533 29670426

[B61] Szelecz I Feddern N Seppey CVW Amendt J Mitchell EAD (2018) The importance of *Saprinus semistriatus* (Coleoptera: Histeridae) for estimating the minimum post-mortem interval. Legal Medicine 30: 21–27. 10.1016/j.legalmed.2017.10.011 29145003

[B62] Tishechkin AK Lackner T (2017) Revision of the type material of the Saprininae and Histerinae (Coleoptera: Histeridae) described by V.O. Kozminykh. Russian Entomological Journal 26(4): 313–317. 10.15298/rusentj.26.4.03

[B63] Wolff M Uribe A Ortiz A Duque P (2001) A preliminary study of forensic entomology in Medellin, Colombia, Forensic Science International 120: 53–59. 10.1016/S0379-0738(01)00422-4 11457610

[B64] Zaatari M (2018) Ex-husband confesses to killing woman found on Sidon beach. The Daily Star. https://www.dailystar.com.lb/ArticlePrint.aspx?id=433382andmode=print

[B65] Zhou Yu-L Caterino MS Ren D Ślipiński A (2020) Phylogeny and evolution of Mesozoic and extant lineages of Histeridae (Coleoptera), with discovery of a new subfamily Antigracilinae from the Lower Cretaceous. Cladistics 2020: 1–19. 10.1111/cla.12418 34618954

